# TREK1 Channels Shape Spindle-Like Oscillations, Neuronal Activity, and Short-Term Synaptic Plasticity in Thalamocortical Circuits

**DOI:** 10.1523/JNEUROSCI.0432-24.2025

**Published:** 2025-09-25

**Authors:** Afsaneh Labbaf, Valérie Krauth, Nicole Rychlik, Venu Narayanan Naik, Laura Vinnenberg, Elif Karabatak, Audrey Teasley, Paula P. Perissinotti, John A. White, Sven G. Meuth, Gilles van Luijtelaar, Francisco J. Urbano, Thomas Budde, Mehrnoush Zobeiri

**Affiliations:** ^1^Institute of Physiology I, University of Münster, Münster D-48149, Germany; ^2^Department of Neurology, Institute of Translational Neurology, University of Münster, Münster D-48149, Germany; ^3^Department of Neurology, Medical Faculty and University Hospital Düsseldorf, Heinrich Heine University Düsseldorf, Düsseldorf D-40225, Germany; ^4^Department of Biomedical Engineering, Center for Systems Neuroscience, Neurophotonics Center, Boston University, Boston, Massachusetts 02215; ^5^Department of Physiology, Molecular and Celular Biology, “Prof. Dr. Héctor Maldonado”, Instituto de Fisiología, Biología Molecular y Neurociencias (IFIBYNE-UBA-CONICET), Facultad de Ciencias Exactas, Universidad de Buenos Aires, Buenos Aires C1428EGA, Argentina; ^6^Donders Centre for Cognition, Radboud University, Nijmegen 6525 XZ, The Netherlands

**Keywords:** KCNK2, sleep, thalamocortical system, thalamus, TREK1

## Abstract

Although TREK1 channels are widely expressed in several thalamic nuclei, the role of this K_2P_ family member in modulating thalamic cell excitability and physiological thalamocortical oscillatory activity is not well studied. Here we explored the contribution of TREK1 channels to membrane properties of two important building blocks of the thalamocortical (TC) system, namely, GABAergic neurons of reticular thalamic nucleus (RTN) and TC neurons in different sensory thalamic nuclei including the ventrobasal complex (VB; somatosensory system) and the medial geniculate nucleus (MGN; auditory system), using male TREK1 knock-out (TREK1^−/−^) mice. Furthermore, we show that the loss of TREK1 channels has distinct effects on neuronal function in different thalamic nuclei. Compared with controls, TREK1^−/−^ mice exhibit decreased excitability in RTN neurons, while VB neurons maintain similar excitability levels. Additionally, the absence of TREK1 channels alters the action potential (AP) characteristics in VB TC neurons and affects GABAergic inhibitory tone in RTN neurons. In TREK1^−/−^ mice, the excitability of cortical pyramidal cells is increased. It is tempting to assume that this combination of changes contributes to a high number of sharp, spindle-like oscillations observed in sleep local field potential (LFP) recordings of these mice. In addition, TREK1^−/−^ mice show a lower amount of delta (1–4 Hz) oscillations during slow-wave sleep and a time-of-day-dependent alteration in the amount of sleep and wakefulness. They also show disturbed auditory signal processing and altered excitability in the auditory thalamus. These findings underline the relevance of TREK1 channels’ broad contribution to the thalamus and thalamocortical system.

## Significance Statement

Using a genetic knock-out approach, we explored TREK1 channels’ impact on the thalamocortical system, focusing on the thalamic reticular nucleus (RTN), ventral medial geniculate nucleus (vMGN), and ventrobasal (VB) nuclei. (1) TREK1 loss altered short-term synaptic plasticity. (2) RTN neuron excitability decreased, vMGN activity increased, and VB excitability remained unchanged, suggesting cell-specific roles and compensatory mechanisms. (3) Network changes included modified slow oscillations, abnormal spindle waves, and heightened auditory responses. (4) Behaviorally, TREK1 loss affected NREM sleep and wakefulness duration. These findings highlight TREK1's critical role in thalamic function.

## Introduction

TREK1 channels belong to the family of two-pore domain K^+^ (K_2P_) channels. As a leak K^+^ conductance, TREK1 is constitutively open at rest and therefore contributes to the maintenance of a hyperpolarized resting membrane potential (RMP). Additionally, the increase in voltage-dependent outward rectification that happens upon membrane depolarization allows the channel to control neuronal excitability and define the duration of APs. The subcellular localization of the channel to both pre- and postsynaptic sites enables TREK1 to participate in neurotransmitter release and synaptic transmission. The channel is activated by different physical and chemical stimuli including but not limited to stretch, heat, pH, polyunsaturated fatty acids, and volatile general anesthetics. In addition, activation of G_s_- and G_q_-protein-coupled membrane receptors (GPCRs) inhibits the channel activity. Therefore, TREK1 channels act as a signal integrator to a wide range of physiological and pathological inputs and, as a consequence, play an important role in the cellular mechanisms of neuroprotection, anesthesia, pain, epilepsy, and depression (Honoré, 2007).

In mammals, TREK1 is highly expressed in different brain regions including the prefrontal cortex, hippocampus, and thalamus. In the somatosensory thalamocortical system, TREK1 is functionally expressed in both RTN and some sensory thalamic nuclei including the VB and the dorsal part of the lateral geniculate nucleus (dLGN; Talley et al., 2001; Bista et al., 2012, 2015; Schroeter et al., 2023). Furthermore, TREK1 channels are also reported to be expressed in thalamocortical afferents of layer IV of the neocortex and auditory TC neurons of the MGN (Talley et al., 2001). However, the neuronal network function of TREK1 in the thalamus, especially in RTN, and the auditory thalamus has not been fully understood. The reciprocal interaction of reticular and specific thalamic nuclei underlies different thalamocortical oscillations including delta activity (1–4 Hz) and sleep spindles (9–12 Hz). Increases in concentration of neurotransmitters (e.g., ACh, NA or 5-HT afferents arising from the ascending brainstem reticular formation) at thalamic and cortical levels have been shown to inhibit TASK1 and TREK1 and activate hyperpolarization-activated and cyclic nucleotide-gated K^+^ channels (HCN channels; Bista et al., 2015; Rychlik et al., 2023). Such inhibition would result in membrane depolarization and a functional switch from burst to tonic firing. It is noteworthy that bursting is the activity mode of TC relay neurons during sleep and anesthesia, while tonic firing is assigned to wakefulness and rapid-eye-movement (REM) sleep. Activation of K_2P_ channels by inhalation anesthetics like isoflurane and halothane results in hyperpolarization of TC relay neurons and therefore increased burst activity (Budde et al., 2008). TREK1 knock-out mice (TREK1^−/−^) show significantly lower sensitivity to these inhalation anesthetics (Heurteaux et al., 2004), although a recent report has questioned that finding (Spencer et al., 2023).

Therefore, it is suggested that K_2P_ channel expression in TC networks is important for the thalamocortical oscillations and for the regulation of the sleep-wake cycle by having an influence on the occurrence of non-rapid-eye-movement (NREM) sleep (Coulon et al., 2012). However, the contribution of TREK1 channels to the latter remains to be elucidated. Moreover, considering the presence of TREK1 in different parts of the TC circuit, it is expected that these channels contribute to the generation of physiological and pathological TC oscillations.

In the present study, we combined in vitro and in vivo recording techniques on TREK1^−/−^ mice to show how TREK1 channels contribute to the passive and active membrane properties of TC neurons. Furthermore, we demonstrate how TREK1 channels affect neuronal excitability and inhibitory GABAergic tone from RTN neurons. Finally, yet importantly, we show how TREK1 deficiency influences physiological TC oscillations, sleep and wake behaviors, and auditory processing.

## Materials and Methods

### Ethics approval

All animal experiments were carried out in accordance with the European Council Committees Directive (2010/63/EU of the European Parliament) and were approved by the local authority (Landesamt für Natur, Umwelt und Verbraucherschutz; AZ: 84-02.04.2015.A574; 81-02.05.50.19.007; 81-02.04.2018.A266). Efforts were made to minimize the number of animals and the degree of discomfort to animals used in this study.

### Mice

Depending on the experimental needs, experiments were performed on 4–14-week-old male C57BL/6J (Envigo) and TREK1^−/−^ mice. TREK1^−/−^ mice were bred in-house (ZTE, University of Münster). Due to the strong influence of the estrous cycle and sex on sleep–wake behavior (Swift et al., 2024) and the smaller effect size found for some aspects of the TREK1^−/−^ phenotype in female mice (Xie et al., 2024), we only used male animals here. All animals were kept under controlled environmental conditions (22–24°C; 50–60% humidity; 12 h light/dark cycle).

### Preparation of acute brain slices for whole-cell patch-clamp recordings

Animals were sacrificed according to effective German legal standards without anesthesia using DecapiCones (Braintree Scientific), and brain tissue was rapidly removed from the skull. Coronal brain slices (250 µm) were prepared in ice-cold oxygenated slicing solution, containing the following (in mM): 200 sucrose, 20 PIPES, 2.5 KCl, 1.25 NaH_2_PO_4_, 10 MgSO_4_, 0.5 CaCl_2_, 10 dextrose, pH 7.35, with NaOH. Before electrophysiological recordings, slices were transferred and kept in a chamber with ACSF (content in mM: 120 NaCl, 2.5 KCl, 1.25 NaH_2_PO_4_, 22 NaHCO_3_, 2 MgSO_4_, 2 CaCl_2_, 25 glucose) at 32°C for 30 min. Thereafter, slices were allowed to cool down to room temperature. The pH was adjusted to 7.35 by bubbling with carbogen (95% O_2_ and 5% CO_2_).

### Whole-cell current-clamp recordings in acute brain slices

The AP firing patterns and membrane properties of TC VB neurons, RTN, and cortical pyramidal neurons in the somatosensory cortex (S1), such as RMP and input resistance (*R*_in_), were analyzed using current-clamp recordings in 4–8-week-old C57BL/6J (wild-type control) and TREK1^−/−^ mice. Recordings were carried out in ACSF containing the following (in mM): 120 NaCl, 2.5 KCl, 1.25 NaH_2_PO_4_, 22 NaHCO_3_, 25 C_6_H_12_O_6_, 2 MgSO_4_, 2 CaCl_2_, 10 glucose, pH 7.25 (adjusted and maintained with continuous bubbling with carbogen) at the temperature of 33 ± 0.5°C. The internal pipette solution contained the following (in mμ): 10 NaCl, 88 K-gluconate, 20 K_3_-citrate, 10 HEPES, 3 BAPTA, 15 phosphocreatine, 3 Mg-ATP, 0.5 Na-GTP, 1 MgCl_2_, 0.5 CaCl_2_ with a pH of 7.25 and an osmolarity of 290–300 mOsmol/kg (Budde et al., 2008; Bista et al., 2012).

The protocol consisted of a series of hyperpolarizing and depolarizing pulses (−250 to 430 pA) with an increment of 20 pA from both RMP and after adjusting the RMP to −55 mV. The length of each pulse was 1 s. No negative or positive current was injected during the measurement of the RMP. *R*_in_ for RTN neurons was determined by calculating the slope from a linear fit to voltage–current (*V*–*I*) curves recorded under current-clamp conditions for each cell. Subthreshold current steps ranging from −90 to +90 pA were injected, and membrane potential (*V*_m_) deflections were measured at the end of each square pulse, discarding any trace that presented action potentials (APs). For VB neurons, *R*_in_ was calculated from the RMP. To characterize and compare APs across groups, dynamic changes in these events were analyzed through phase plot analysis (Trombin et al., 2011; Fohlmeister, 2015). For each action potential, the derivative of the membrane potential with respect to time (dV/dt, expressed in mV/ms) was calculated and plotted against the corresponding instantaneous membrane potential (mV). The phase plot and corresponding AP characteristics, including AP amplitude, half-width, afterhyperpolarization (AHP) amplitude, threshold, and maximum depolarization and repolarization slopes, were analyzed using Clampfit 7.10 for threshold APs evoked from a membrane potential of −55 mV.

### Synaptic current recordings in acute brain slices

Synaptic properties were assessed in TC neurons recorded from VB and RTN of 4–8-week-old wild-type (WT) and TREK1^−/−^ mice. Evoked inhibitory postsynaptic currents (IPSCs) were recorded in an external solution containing the following (in mM): 120 NaCl, 2.5 KCl, 1.25 NaH_2_PO_4_, 22 NaHCO_3_, 25 C_6_H_12_O_6_, 2 MgSO_4_, 2 CaCl_2_, 10 glucose and in the presence of glutamate receptor blockers AP-5 (20 μM, Tocris) and DNQX (10 μM, Tocris). Spontaneous (sIPSCs) and miniature IPSCs (mIPSCs) were recorded in VB neurons. Tetrodotoxin (TTX, 1 µM, Abcam) was used for recording mIPSCs. The patch pipettes were filled with an internal solution containing the following (in mM): 110 CsCl, 20 HEPES, 10 TEA-Cl, 12 phosphocreatine, 2 Mg-ATP, 0.5 Na-GTP, 1 MgCl_2_, adjusted to pH 7.3 with CsOH. All recordings were performed by patching the soma of TC neurons and using an EPC-10 amplifier (HEKA Elektronik). The access resistance was in the range of 5–25 MΩ and was monitored throughout the experiment. Cells with an access resistance of >25 MΩ were discarded from the experiment. Series resistance compensation of >30% was routinely applied. Analysis of IPSCs was done using Clampfit 10.7 and Origin 2021 software. Amplitudes and interevent intervals were fitted with a sigmoid fit, and median values were used for comparison between groups. Mini Analysis software (Synaptosoft, version 6.0.8) was used to analyze rise and decay time, half-width, and charge transfer based on the event count per cell (Studtmann et al., 2023). For analysis, the data were digitally filtered at 1 kHz. Events were automatically detected when the amplitude was at least three times higher than the root mean square (RMS) noise level. Data are displayed as scatterplots, showing individual cell data points along with the group mean, accompanied by SEM on the left *y*-axis. The mean or median (for non-normally distributed datasets) difference was plotted on the right *y*-axis.

Paired-pulse inhibitory postsynaptic currents (eIPSCs) were recorded in VB neurons while being evoked with an interstimulation interval of 100 ms using bipolar tungsten electrodes (MicroProbes) positioned at the boundary between RTN and VB. To block Na^+^ currents and avoid postsynaptic APs, 10 mM *N*-(2,6-dimethylphenylcarbamoylmethyl) triethylammonium chloride (QX-314, Alomone labs) was added to the pipette solution (Urbano et al., 2009; Goitia et al., 2016). Bicuculline (20 µM, Tocris) was used to confirm that eIPSC currents were mediated by GABA_A_ receptors. In some experiments, the effect of spadin, a potent blocker of TREK1 channels, on eIPSCs was assessed. After initial recordings of eIPSCs under control conditions, slices were incubated with synaptic blockers and 10 µM spadin for 30 min to ensure its effective delivery to the axon terminals and complete blockade of TREK channels.

### Voltage–current (*V*–*I*) recording of RTN in the presence of synaptic blockers in acute brain slices

This series of experiments was conducted on RTN neurons in the presence of ionotropic, metabotropic, and synaptic receptor blockers [2 µM CGP-55845 hydrochloride, 10 µM bicuculline, 10 µM DNQX, 20 µM AP-5, and 50 µM trans-ACPD (all from Tocris)]. These blockers were used to isolate the neurons’ subthreshold intrinsic properties and to suppress both spontaneous and evoked synaptic activity during experiments, enabling the quantification of subthreshold *V*–*I* curves and *R*_in_ values under current-clamp conditions.

### In vivo LFP recording

Twelve- to fourteen-week-old male C57BL/6J mice (*n* = 5) and TREK1^−/−^ (*n* = 5) were used as control and experimental groups, respectively. Implantation of the LFP recording electrodes, data collection, and signal analysis were performed as previously described (Zobeiri et al., 2018). Briefly, animals were anesthetized with an intraperitoneal (i.p.) injection of 50 mg/kg pentobarbital supplemented by a subcutaneous injection of carprofen (Rimadyl; 5 mg/kg). The mouse head was fixed in a stereotactic frame (David Kopf Instruments), and holes were drilled into the skull on top of the right hemisphere. Isolated (except at the tip) stainless steel wire recording electrodes (diameter, 0.127 mm; impedance, 500 KΩ; Franco Corradi) were inserted in the primary somatosensory cortex (S1; A/P= 0, M/L = 3, depth = −1.2), ventral posterior medial nucleus of the thalamus (VPM, A/P = −1.7, M/L = 1.5, depth = −2.8), reticular thalamic nucleus (RTN, A/P = −0.7, M/L = 1.2, depth = −3), and two epidural silver wires were placed on top of the cerebellum to serve as ground and reference electrodes. The electrode assembly was fixed to the skull with the aid of dental acrylic cement. Following surgery, mice were single-housed and allowed to recover for at least 1 week. LFP signals were recorded continuously for 24 h from 8 A.M. (with lights off at 8 P.M.). Animals were connected to the recording setup via a cable and swivel allowing them to move freely during the recording. The LFP signals were amplified (Science Products DPA-2F), filtered by a bandpass filter with cutoff points at 1 Hz (high-pass) and 100 Hz (low-pass) and digitalized with a constant sampling rate of 260 Hz by a CED recording system (Cambridge Electronic Design). The behavioral activity of the animals was registered by a Passive Infrared Registration System (PIR, RK2000DPC LuNAR PR Ceiling Mount, Rokonet RISCO Group) placed on top of the registration box (van Luijtelaar et al., 2012). Following LFP recordings, animals were deeply anesthetized by isoflurane, and brains were removed for histological verification of the correct electrode position. Offline analysis of recordings was carried out using NeuroExplorer 5 (Nex Technologies) and Spike2 (version 7.08, Cambridge Electronic Design) software. Power spectral density (PSD) was calculated for 128 bins covering the 1–100 Hz frequency range (delta, 1–4 Hz; theta, 4.5–8 Hz; alpha, 8–12; beta, 12.5–30; gamma, 30.5–100) with a bin width of 0.78 Hz. The PSD data from 30 epochs of 2 s for active-wakefulness and 30 epochs of 10 s for slow-wave sleep were averaged for each individual animal. Epochs of active-wakefulness and deep slow-wave sleep were selected based on the LFP and PIR activity as previously described (Zobeiri et al., 2018). The NREM sleep selection criteria included high-amplitude cortical electroencephalography (EEG) together with slow (1–4 Hz) waves in a motionless animal (as established by the PIR signal). Active-wakefulness criteria included behavioral activity (detected by high and variable PIR signal) accompanied by low-amplitude cortical EEG with theta and beta frequency oscillations. Only epochs from the first 2 h of the light period were selected; these hours represent the periods with the highest amount of specifically deep slow-wave sleep (Huber et al., 2000). To control individual differences in LFP amplitude, relative values (PSD%) were analyzed. Differences in PSD values between WT and TREK1^−/−^ mice for each state of vigilance were evaluated by separate mixed-repeated-measures analyses of variance (ANOVAs) followed by post hoc Student's *t* tests (two sided).

For analysis of sleep/wake cycles, different states of vigilance including NREM, REM, and wakefulness (both active and passive) were scored manually by a trained individual. The selection criteria for NREM and wakefulness were as described earlier. The REM sleep criteria included low-amplitude cortical theta like wake LFP, however, accompanied by a flat PIR. Sleep–wake scoring was performed on the first 10 min of every hour from the 24 h LFP recordings (total of 240 min LFP data per animal). Analysis of sleep spindles during NREM was performed on visually defined spindles which were detected after filtering the raw LFP signal with two separate filters: 1–4 Hz for detection of delta and 10–15 Hz for spindle oscillations.

### In vivo unit activity recording

The spontaneous and auditory-induced unit activities in freely behaving mice were recorded with microwire arrays (one array, eight electrodes, and one reference/array per brain region; Stablohm 650; California Fine Wire). The arrays were implanted under stereotaxic surgery (David Kopf Instruments) under deep pentobarbital (50 mg/kg) anesthesia, supplemented by subcutaneous injection of carprofen (Rimadyl; 5 mg/kg). All pressure points were covered with 2% xylocaine gel (Astra Zeneca) and the tissue to be incised was injected with 2% xylocaine solution. The electrode arrays were implanted in the left hemisphere at specific stereotaxic coordinates: layer IV of the primary auditory cortex (AC1): A/P: −2.18 mm; M/L: −4.2 mm from bregma; and depth: −1 mm from the brain surface; or in the ventral part of the medial geniculate nucleus (vMGN; A/P: −3.16 mm; M/L: −2.2 mm; depth: −2.90 mm; Paxinos and Franklin, 2001).

After 7–10 d of surgical recovery, unit activities were recorded. Recordings were performed before, during, and after the presentation of an auditory stimulus consisting of a sequence with six repetitions of either low- or high-frequency tones [pseudorandomized; 2.5 and 10 kHz at 85 dB; for the protocol see Cerina et al., 2017 (Fig. 11)]. Neuronal activity was recorded with a Multichannel Amplifier System (Alpha Omega). Unit activities were bandpass filtered at 100 Hz to 20 kHz, with a sampling rate of 40 kHz. Spikes of individual neurons were sorted by time–amplitude window discrimination and principal component analysis (Offline Sorter, Plexon) and verified through quantification of cluster separation, as described before (Narayanan et al., 2018).

Basal and stimulus-evoked activities, as well as *z*-scores of sorted neurons, were analyzed by a customized MATLAB routine (The MathWorks). The time axis of experimental sessions was divided into 1 s bins to calculate firing rates and their *z*-scores. Firing rates have been calculated as AP count per second. Values obtained during the first 60 s of an experimental session were defined as baseline activity for every single neuron. Individual firing rates were *z*-scored to their respective mean baseline activity. Neurons were defined as “ON” neurons if at least one bin showed a *z*-score ≥1.96 in response to stimulus presentation. Since the TREK1 deletion affects the overall firing activity, the firing latencies were also analyzed. The latency was defined as the time between the stimulus presentation and the neuronal response in the AC1 or MGN. In the narrative, *n* numbers are given as the number of recorded neurons.

### Real-time quantitative PCR

For RNA isolation and reverse transcription tissues were freshly prepared from different brain regions of mice. Collected tissue was stored in RLT lysis buffer with β-mercaptoethanol until further use at −80°C. Total RNA was isolated from the dissected brain tissue with RNeasy Micro kit (Qiagen). Isolated RNA was transcribed into cDNA using Superscript III reverse transcriptase (Thermo Fisher Scientific) for 1 h at 37°C. Amplification of cDNA was carried out with Mastercycler realplex4 (Eppendorf), including the following steps: 10 min at 95°C, 15 s at 95°C, and 1 min at 60°C, with a repeat of 40 times for the second and third steps. Taqman primers with FAM (Thermo Fisher Scientific, Scn1a: Mm00450580_m1, Scn3a: Mm07297464_m1, Scn8a: Mm00488110_m1, Trpm4: Mm00613173_m1, TREK2: Mm00504118_m1, Gabra1: Mm00439046_m1, Gabrb2: Mm00433467_m1, Gabrg2: Mm00433489_m1, Gabra4: Mm00802631_m1, Gabrd: Mm01266203_g1) were used for the genes to be investigated. Data were normalized against the endogenous housekeeping gene GAPDH (Mm99999915_g1).

### Computational modeling

The simulations presented here were conducted using a modified thalamic network model (Destexhe et al., 1996) in the NEURON simulation environment, as previously described (Oniani et al., 2022; Vinnenberg et al., 2024). The model simulates delta oscillations by connecting two TC VB and two RTN neurons via AMPA and GABA_A+B_ synapses. The potassium leak conductance of the two TC and RTN neurons was decreased incrementally by 1% of their initial value, up to −15%. In these simulations, the interburst frequency between the first and second bursts, the number of action potentials within the first burst, and the time to the first spike within the first burst of each TC cell were measured.

### Statistical analysis

All data are expressed as the mean ± standard error of the mean. Student's *t* tests were used for simple comparison between groups. In case of multiple comparisons, one-way or repeated-measures ANOVAs were used. Mauchly's test of sphericity was used for the repeated-measures ANOVA. Where sphericity assumption was violated (*p* < 0.05), the Greenhouse–Geisser correction was applied. Student's *t* tests were used as post hoc tests. In addition, Kolmogorov–Smirnov test (KS test) was used to compare cumulative curves of GABAergic mIPSC/sIPSC currents. An unpaired *t* test with Welch's correction was applied to compare the rise time, decay time, half-width, and charge transfer of mIPSCs in both VB and RTN neurons for normally distributed data. For non-normally distributed data, a Mann–Whitney *U* test was used. The mean or median difference was calculated as the difference between TREK1^−/−^ and WT cells.

The statistical analysis was performed using IBM SPSS Statistics for Windows, version 27.0 (IBM) and GraphPad Prism, version 7.05. Origin, version 2020b (OriginLab) and CorelDRAW Graphics Suite X8 were used for figure plotting. Differences were considered statistically significant if *p* values were <0.05 (*, **, or ***, ****, indicate *p* < 0.05, *p* < 0.01, *p* < 0.001, or *p* < 0.0001, respectively).

## Results

### TREK1 channels regulate membrane properties in RTN but not VB TC neurons

**Figure 1.**
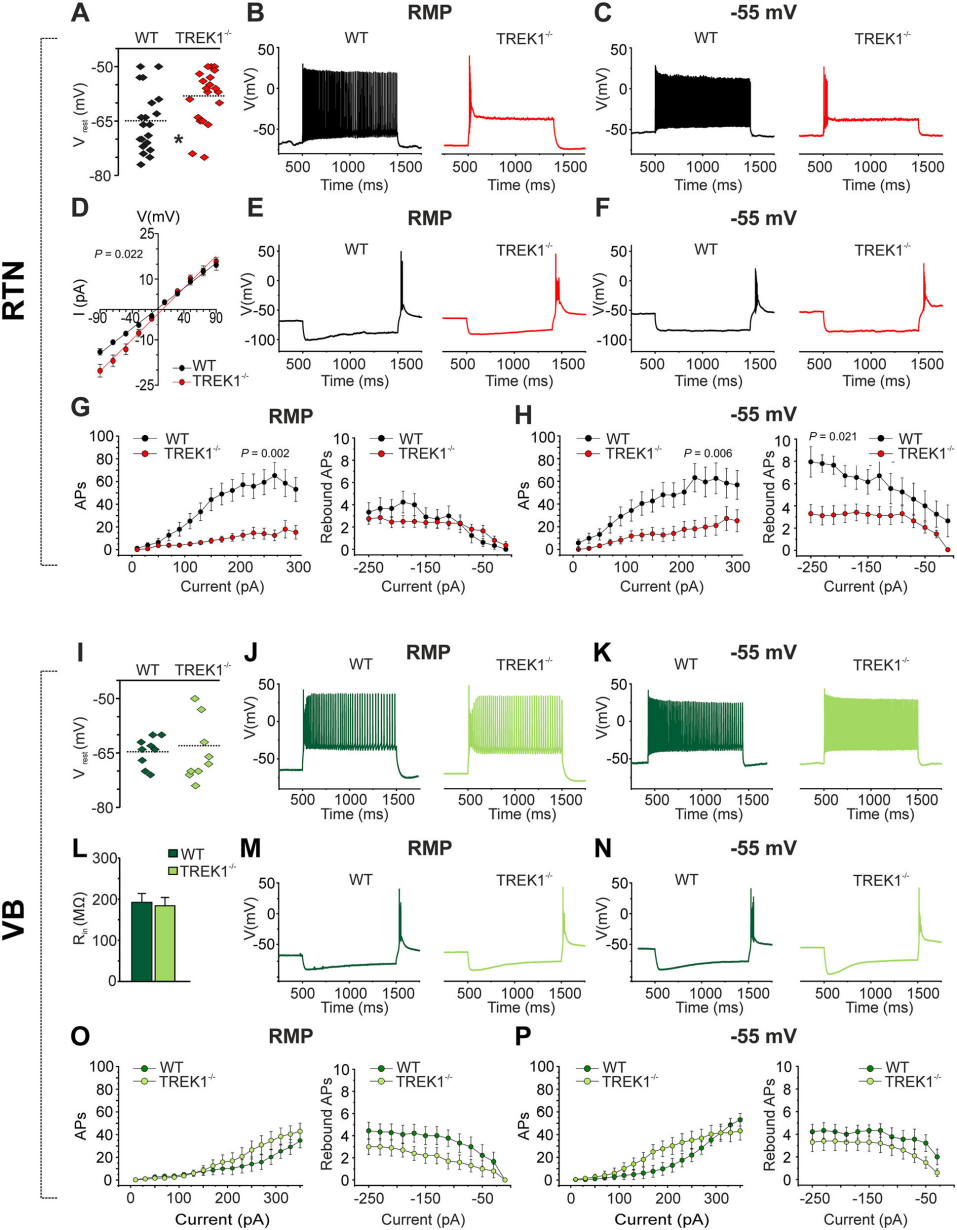
TREK1 channels regulate membrane properties and firing pattern of RTN neurons. ***A***, Graph compares the resting membrane potential (*V*_rest_) of RTN neurons between wild-type (WT) and TREK1^−/−^ mice. ***B***, ***C***, ***E***, ***F***, Representative traces of action potential (AP) firing elicited by depolarizing (1 s duration; +350 pA) and hyperpolarizing (1 s duration, −250 pA) current pulses from RMP and after adjusting the RMP to −55 mV in RTN neurons of WT (black trace) and TREK1^−/−^ mice (red trace). ***D***, Graph compares the mean voltage–current (*V*–*I*) curves of RTN neurons between WT and TREK1^−/−^ mice. ***G***, ***H***, Graphs compare the number of APs elicited by injection of positive currents with 20 pA increments from RMP (***G***) and from −55 mV (***H***). A significantly smaller number of APs was evoked in TREK1^−/−^ mice compared with WT both from RMP and −55 mV. Comparison of the number of APs on rebound burst between WT and TREK1^−/−^ showed significant differences only at −55 mV. ***I***, Graph compares *V*_rest_ of ventrobasal thalamocortical relay (VB TC) neurons in wild-type (WT) and TREK1^−/−^ mice. ***J***, ***K***, ***M***, ***N***, Representative traces of AP firing elicited by depolarizing (1 s duration; +350 pA) and hyperpolarizing (1 s duration, −250 pA) current pulses from RMP and after adjusting the RMP to −55 mV in VB TC neurons of WT (in dark green) and TREK1^−/−^ mice (in light green). ***L***, Graph compares the *R*_in_ of VB TC neurons between WT and TREK1^−/−^ mice. ***O***, ***P***, Graphs compare the number of APs elicited by injection of positive currents with 20 pA increments from RMP (***O***) and from −55 mV (***P***). No significant difference in the number of APs was observed between TREK1^−/−^ and WT VB TC neurons at both RMP and −55 mV.

To assess the functional impact of TREK1 channels on passive and active membrane characteristics of RTN neurons, we performed a whole-cell current-clamp experiment on somatosensory RTN neurons of WT control and TREK1-deficient mice (Fig. 1). The protocol consisted of series of hyperpolarizing and depolarizing pulses of defined amplitude and duration (see Materials and Methods section). Compared with WT mice, RTN neurons in TREK1^−/−^ mice showed a significantly more depolarized RMP (Fig. 1*A*; −65.0 ± 1.9 mV, *n* = 20 cells WT vs −58.4 ± 1.7 mV, *n* = 20 cells, TREK1^−/−^, *t* = 2.6, df = 38, *p* = 0.014) and higher slope of *V*–*I* curve (i.e., mean *R*_in_; Fig. 1*D*, 170 ± 12 MΩ, *n* = 16 cells WT vs 221 ± 17.4 MΩ, *n* = 17 cells TREK1^−/−^, *t* = 2.42, df = 28.11, Welch's *t* test, *p* = 0.022), thereby pointing to less active conductances in knock-out mice. Comparing the slope of mean *R*_in_ between RTN neurons of WT control and TREK1 KO in the presence of ionotropic, metabotropic, and synaptic blockers, no significant changes were observed (data not shown; 237 ± 54.5 MΩ, *n* = 5 cells WT vs 183 ± 32 MΩ, *n* = 9 cells TREK1^−/−^, *t* = 0.85, df = 6.82, Welch's *t* test, *p* = 0.42), thereby indicating differential influences of synaptic activity in both genotypes. Moreover, the number of APs triggered by injection of depolarizing pulses from RMP was significantly lower in TREK1^−/−^ RTN neurons (repeated-measures ANOVA, test of within subject effects showed a significant interaction between depolarization steps and genotypes, *F*_(2.5,101.9)_ = 6.92, *p* < 0.001; test of within subject factor showed significant main effect for genotypes: *F*_(1,38)_ = 11.7, *p* = 0.002, Student's *t* test showed significant differences in steps from 110 to 310 pA, *p* < 0.05) when compared with those of WT mice (Fig. 1*B*,*G*). Interestingly, even after adjusting the RMP in both groups to a similar membrane potential (holding potential of −55 mV), the number of APs still remained significantly lower in TREK1^−/−^ RTN neurons compared with WT mice (Fig. 1*C*,*H*; test of between subject factor showed significant main effect for genotypes: *F*_(1,38)_ = 8.6, *p* = 0.006). In addition to K_2P_ channels, the hyperpolarization-activated potassium inward current (*I*_h_) generated by HCN2 channels in mice RTN is important in regulation of RMP at a somatic/dendritic level (Meuth et al., 2006; Bista et al., 2012). Comparing the *I*_h_-induced voltage sag between WT and TREK1^−/−^ RTN neurons did not reveal any differences between the two groups (Fig. 1*E*,*F*). In TREK1^−/−^ mice, injection of hyperpolarizing current pulses to RTN neurons from membrane potential of −55 mV elicited significantly lower number of APs on rebound burst (Fig. 1*H*; *F*_(1,38)_ = 5.8, *p* = 0.021).

To assess the role of TREK1 channels in shaping the firing patterns and intrinsic properties of thalamic relay neurons, whole-cell current-clamp recordings were performed on VB TC neurons. Comparison of the passive membrane properties (RMP and *R*_in_) and number of APs elicited by depolarization of VB TC neurons from RMP, as well as from −55 mV, did not reveal any differences between TREK1^−/−^ and WT mice (Fig. 1*I–P*). These findings indicate that knock-out of TREK1 channels differentially affect membrane characteristics and firing pattern in RTN and VB TC neurons, with the latter showing roughly no changes.

### TREK1 channels contribute to shaping AP dynamics in RTN and VB neurons

**Figure 2.**
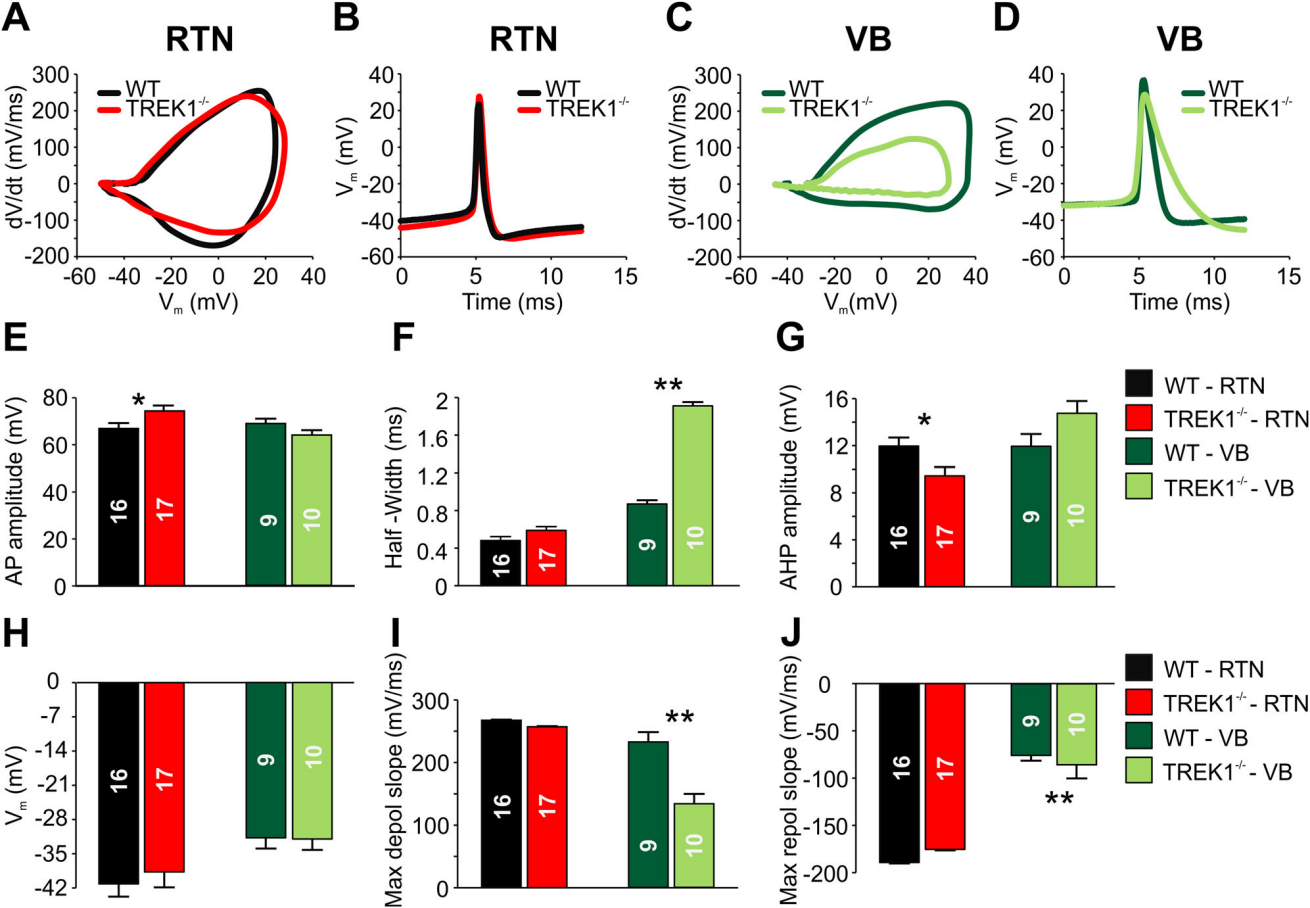
Role of TREK1 in shaping action potential characteristics in RTN and VB neurons. The phase plot and corresponding AP (action potential) characteristics graphs were generated using the threshold AP elicited at a membrane potential of −55 mV. ***A***, Representative phase plots of WT (black) and TREK1^−/−^ (red) RTN neurons. ***B***, Averaged traces of evoked APs recorded in RTN of WT and TREK1^−/−^ mice that were used to generate the phase plot and corresponding AP analysis presented in panels ***E–J***. ***C***, Representative phase plots of WT (dark green) and TREK1^−/−^ (light green) VB TC neurons. ***D***, Averaged traces of evoked APs recorded in VB TC neurons of WT and TREK1^−/−^ mice that were used to generate the phase plot and corresponding AP analysis presented in panels ***E–J***. ***E***, Comparison of AP amplitude between WT and TREK1^−/−^ neurons. ***F***, Comparison of AP half-width between WT and TREK1^−/−^ RTN and VB TC neurons. ***G***, Afterhyperpolarization (AHP) amplitude (mV) of APs in WT and TREK1^−/−^ RTN and VB TC neurons, with AHP measured from the AP threshold to the AHP peak. ***H***, Comparison of AP threshold between WT and TREK1^−/−^ neurons. ***I***, ***J***, Maximum depolarization and hyperpolarization slopes of APs in WT and TREK1^−/−^ RTN and VB TC neurons, respectively. Numbers inside each bar represent the number of cells used for each condition.

In addition to their impact on passive membrane properties, background K^+^ channels are also important in controlling the AP frequency and duration. To investigate the role of TREK1 channels in defining AP dynamics in RTN neurons, phase plots were generated to visualize the averaged threshold AP, i.e., the first AP triggered by the pulse protocol from a holding potential of −55 mV (Fig. 2*A*,*B*). Different properties, including AP amplitude, half-width, AHP amplitude, AP threshold, and maximum depolarization and repolarization slopes, were compared between TREK1^−/−^ and WT RTN neurons (Fig. 2*E–J*). Analysis revealed that TREK1^−/−^ neurons had a significantly higher AP amplitude (Fig. 2*E*; 74.3 ± 2.5 mV, *n* = 17 cells) compared with WT neurons (66.9 ± 2.3 mV, *n* = 16 cells; *t* = 2.6, df = 31, *p* = 0.037) and a lower AHP amplitude (Fig. 2*G*; 9.4 ± 0.9 mV, *n* = 17 cells) compared with WT neurons (11.9 ± 0.73 mV, *n* = 16 cells; *t* = 2.1, df = 31, *p* = 0.039). No significant differences were found in AP threshold of TREK1^−/−^ (−38.7 ± 5.1 mV) and WT (−41.2 ± 1.4 mV) RTN neurons.

Next, phase plot analysis was performed on APs obtained from TREK1^−/−^ and WT VB TC neurons (Fig. 2*C*,*D*). TREK1^−/−^ neurons exhibited a significantly higher half-width (Fig. 2*F*; 1.9 ± 0.3 mV, *n* = 10 cells) compared with WT neurons (0.9 ± 0.4 mV, *n* = 9 cells, *t* = 3.4, df = 17, *p* = 0.007). Additionally, TREK1^−/−^ neurons showed a significantly slower maximum depolarization slope (Fig. 2*I* 133.5 ± 15.7 mV/ms) compared with WT neurons (232.3 ± 15.7 mV/ms, *n* = 9 cells, *t* = 3.2, df = 17, *p* = 0.003) and a significantly slower maximum repolarization slope (Fig. 2*J*; −44.9 ± 7.3 mV/ms, *n* = 10 cells) compared with WT neurons (−75.6 ± 5.6 mV/ms, *n* = 9 cells, *t* = 3.3, df = 17, *p* = 0.004). No differences were found in the AP threshold between WT (−31.7 ± 1.1 mV, *n* = 9 cells) and TREK1^−/−^ (−31.9 ± 2.2 mV, *n* = 10 cells) mice, nor in AHP amplitude (10.9 ± 0.9 mV, *n* = 9 cells for WT vs 13.4 ± 2.3 mV, *n* = 10 cells for TREK1^−/−^). These findings highlight the important role of TREK1 channels in modulating AP characteristics in RTN and VB TC neurons, particularly the shape and kinetics of the action potential, rather than influencing passive membrane properties or firing frequency.

### Effects of acute TREK1 channel block by spadin

**Figure 3.**
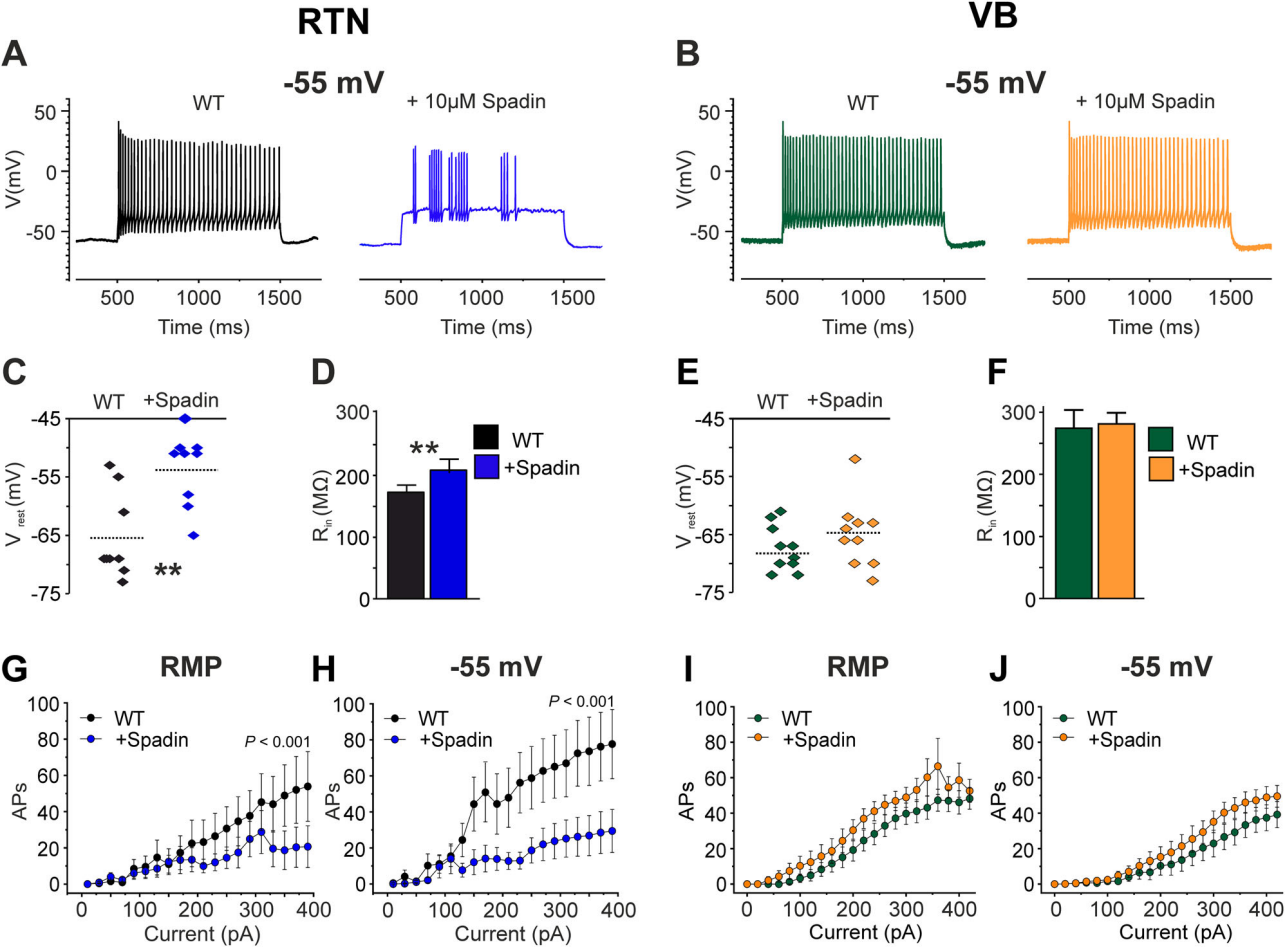
Spadin, a specific TREK1 channel blocker, reduces action potential firing in GABAergic RTN neurons but not glutamatergic VB TC neurons. ***A***, ***B***, Example traces of APs in RTN (***A***) and VB TC neurons (***B***) of WT elicited by depolarizing (1 s duration; +350 and +340 pA in RTN and VB neurons, respectively) current pulses from −55 mV, before and after application of 10 µM spadin, a potent TREK1 inhibitor. ***C***, ***D***, Graphs compare the *V*_rest_ and mean *V*–*I* curves of RTN neurons of WT mice before and after application of 10 µM spadin. ***E***, ***F***, Graphs compare the *V*_rest_ and *R*_in_ of VB TC neurons of WT mice before and after application of 10 µM spadin. ***G, H***, Graphs compare the number of APs elicited by injection of positive currents with 20 pA increments from RMP (***G***) and after adjusting the membrane potential to −55 mV (***H***) in WT RTN neurons in control condition and in presence of 10 µM spadin. A significantly smaller number of APs was evoked after application of spadin in RTN neurons. ***I***, ***J***, Graphs compare the number of APs elicited by injection of positive currents with 20 pA increments from RMP (***I***) and after adjusting the membrane potential to −55 mV (***J***) in WT VB TC neurons in control condition and in presence of 10 µM spadin. Here, spadin could not induce any changes in the number of APs.

To compare TREK1 gene knock-out and ion channel block, the impact of spadin, a specific TREK1 channel inhibitor, was assessed on passive and active membrane properties of RTN neurons in WT mice (Fig. 3). While bath application of 1 µM spadin did not change the firing properties of RTN cells in WT mice (data not shown), 10 µM spadin resulted in a significant depolarizing shift in the RMP (Fig. 3*C*; −65.44 ± 2.4 mV control vs −53.44 ± 2.1 mV +10 µM spadin, *n* = 9 cells, *t* = 4.012, df = 8, *p* = 0.004). No significant change in the slope of mean *R*_in_ was observed between WT mice and following the application of 10 µM spadin (Fig. 3*D*; 182.3 ± 22 MΩ, *n* = 10 cells control vs 141 ± 25 MΩ, *n* = 8 cells, *t* = 1.24, df = 24.6, Welch's *t* test, *p* = 0.22). Additionally, no significant difference was found between conditions in the presence of synaptic blockers (data now shown; 237 ± 54.5 MΩ, *n* = 5 cells control vs 158 ± 27 MΩ +10 µM spadin, *n* = 5 cells, *t* = 1.3, df = 5.85, Welch's *t* test, *p* = 0.24). It should be noted that the spadin-induced depolarization reached potentials that are consistent with the activation of voltage-dependent channels in some neurons. Furthermore, the number of APs elicited by depolarization from both RMP (repeated-measures ANOVA, test of within subject effect, significant interaction of depolarization steps and spadin application, *F*_(21,168)_ = 2.43, *p* < 0.001) and −55 mV (repeated-measures ANOVA, test of within subject effect, significant interaction of depolarization steps and spadin application, *F*_(21,168)_ = 3.62, *p* < 0.001) were significantly reduced in the presence of 10 µM spadin (Fig. 3*G*,*H*). This is while spadin did not affect the membrane properties and firing pattern of TREK1^−/−^ RTN neurons (data not shown). Furthermore, application of 10 µM spadin to block TREK1 channels in WT VB TC neurons had no effect on passive membrane properties or firing behavior (Fig. 3*B*,*E*,*F*,*I*,*J*). Thus, these findings indicate that pharmacological block of TREK1 largely mimics the effect of gene knock-out in thalamic neurons.

### TREK1 deficiency results in alteration of synaptic transmission and GABAergic release from RTN

**Figure 4.**
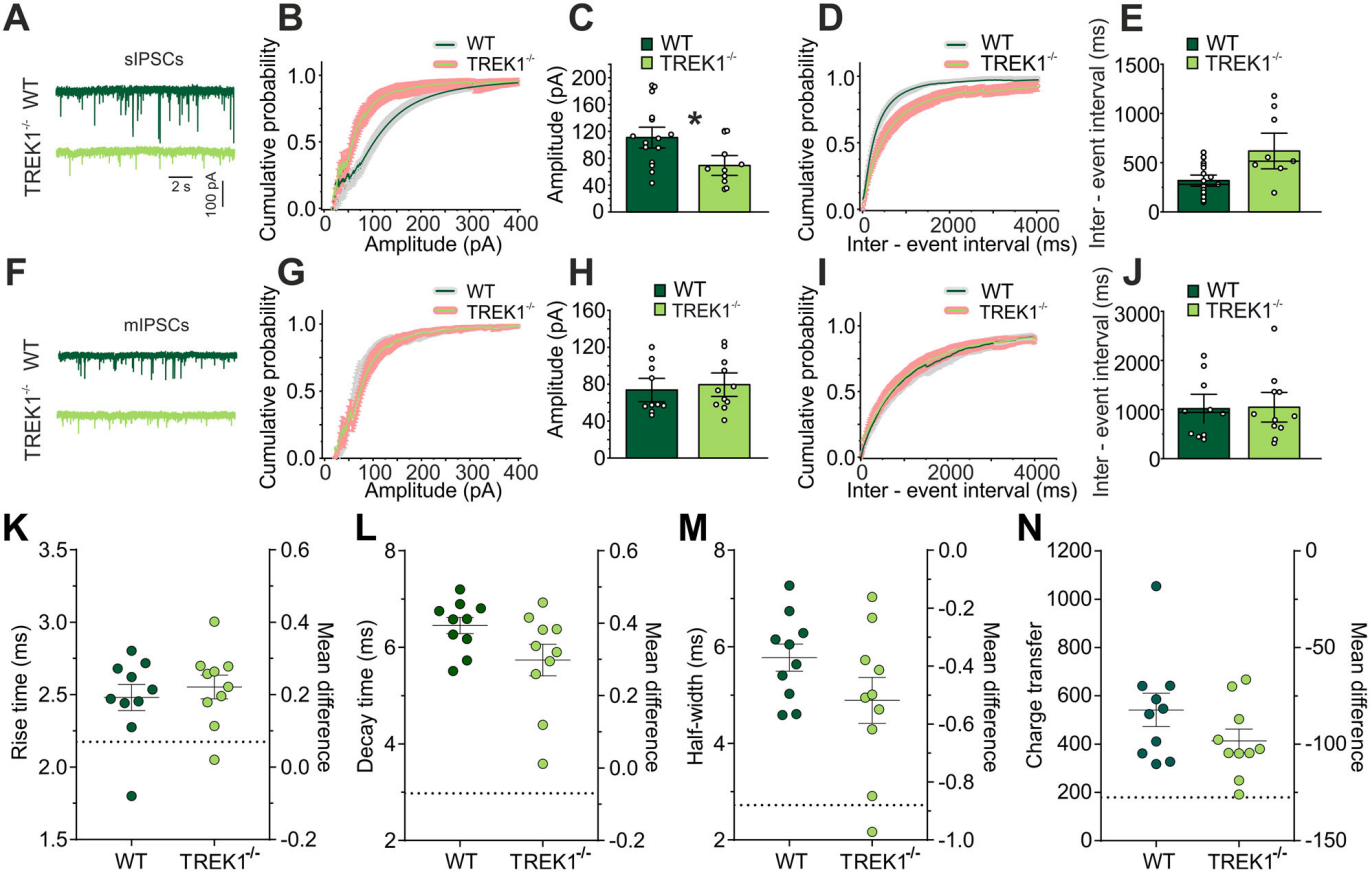
TREK1 channels and GABAergic transmission onto VB TC neurons. ***A***, Example traces of spontaneous inhibitory postsynaptic currents (sIPSCs) recorded in ventrobasal (VB) relay neurons of WT and TREK1^−/−^ mice. ***B***, Representative cumulative probability plots of sIPSCs amplitude recorded in VB relay neurons show a significant negative shift in TREK1^−/−^. ***C***, Bar graph compares the sIPSCs median amplitude between WT and TREK1^−/−^ VB neurons. ***D***, Cumulative probability plots of sIPSCs interevent interval recorded from VB relay neurons of WT and TREK1^−/−^ mice. ***E***, Bar graph compares the sIPSCs median interevent interval between WT and TREK1^−/−^ VB neurons. ***F***, Example traces of GABAergic miniature postsynaptic currents (mIPSCs) recorded in VB relay neurons of WT and TREK1^−/−^ mice. ***G***, Representative cumulative probability plots of mIPSCs amplitude recorded in VB relay neurons. ***H***, Bar graph comparing the mIPSCs median amplitude between WT and TREK1^−/−^ VB neurons. ***I***, Cumulative probability plots of mIPSCs interevent interval recorded from VB relay neurons of WT and TREK1^−/−^ mice. ***J***, Bar graph compares the mIPSCs median interevent interval between WT and TREK1^−/−^ VB neurons. ***K–N***, Scatterplots display rise time, decay time, half-width, and charge transfer for individual cell data points between WT and TREK1^−/−^ VB neurons, with the group mean indicated on the left *y*-axis. The difference between groups mean is shown with a dashed line on the right *y*-axis. Error bars represent the standard error of the mean (SEM).

Bursting in RTN neurons conveys a powerful synaptic inhibition onto thalamic relay cells through activation of GABA_A_ receptors on TC neurons. This can be recorded as large inhibitory postsynaptic potentials (IPSCs) in VB TC cells. The inhibition of TC relay cells is important in generation of burst firing in these neurons and consequently thalamocortical oscillations. To understand how changes in firing pattern and membrane properties of RTN cells might have influenced the synaptic characteristics of TC neurons, spontaneous (sIPSC; Fig. 4*A–E*) and miniatures (mIPSCs; Fig. 4*F–J*) IPSCs were measured in VB TC neurons by whole-cell voltage-clamp recording. The cumulative probability plots of sIPSCs (Fig. 4*B*,*C*) showed a significantly lower amplitude for TREK1^−/−^ VB TC cells compared with those of WT mice (110.8 ± 10.3 pA, *n* = 18 cells, WT vs 69.2 ± 9.9 pA, *n* = 10 cells, TREK1^−/−^, independent-samples Kolmogorov–Smirnov test, *p* = 0.02). The cumulative probability plots of sIPSCs for interevent intervals revealed less occurrence for TREK1^−/−^ VB TC cells (317.43 ± 37.64 ms, *n* = 19 cells, WT vs 618.5 ± 120.6 ms, *n* = 10 cells, TREK1^−/−^; Fig. 4*D*,*E*); however, the higher dispersion of intervals observed in TREK1^−/−^ yielded no statistical significance (independent-samples Kolmogorov–Smirnov test, *p* > 0.05). Next, mIPSCs were recorded in presence of 1 µM TTX (Fig. 4*F*). Comparing the amplitude and frequency of mIPSCs between TREK1^−/−^ and WT VB TC cells did not show any significant differences (Fig. 4*G–J*). Furthermore, no significant differences were observed between TREK1^−/−^ and WT VB TC neurons in mIPSC kinetic properties, including rise time, decay time, half-width, or charge transfer (Fig. 4*K–N*).

**Figure 5.**
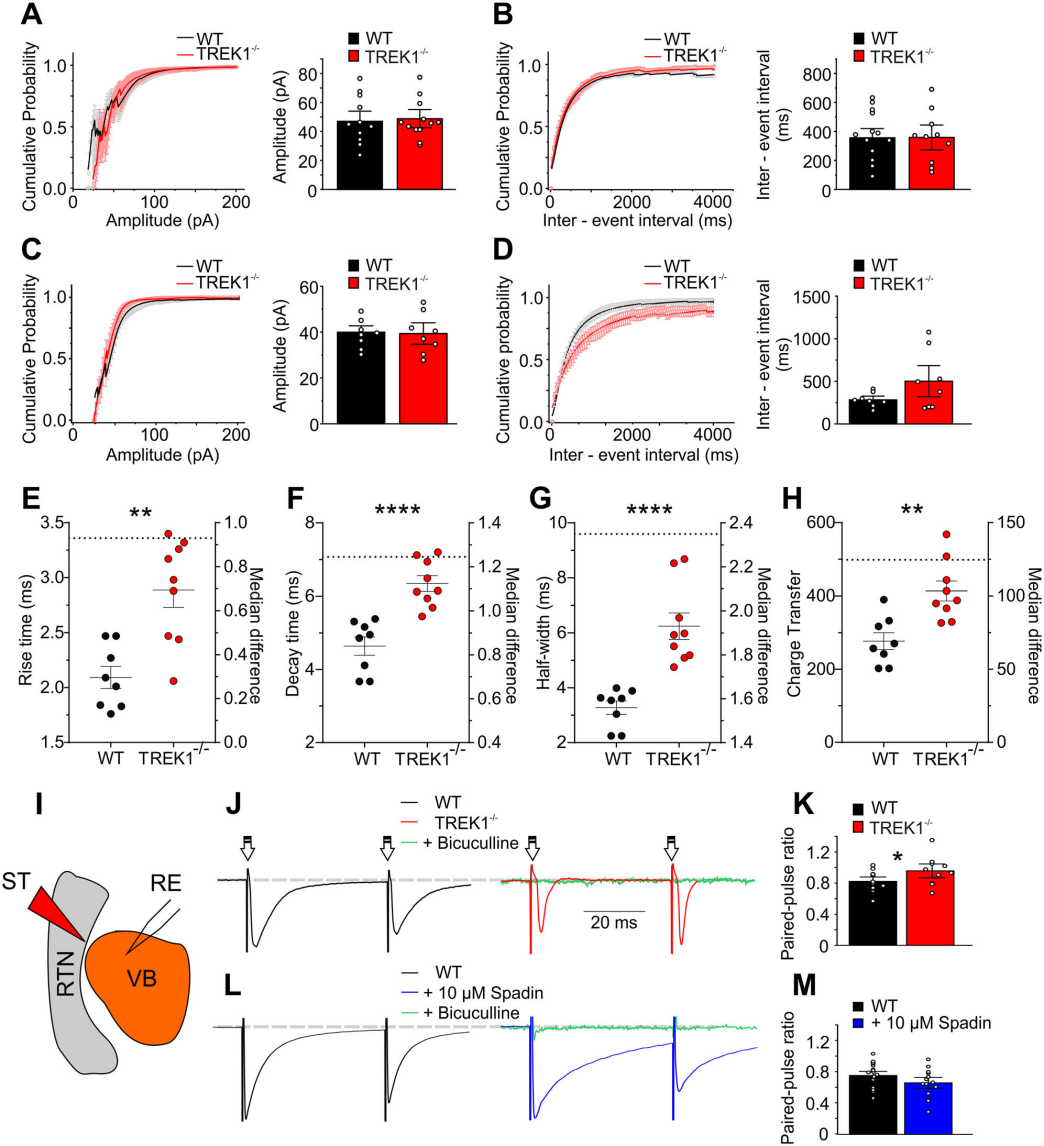
TREK1 channel deficiency alters the evoked GABAergic release probability in RTN. ***A***, Cumulative probability plots of sIPSCs amplitude recorded from GABAergic neurons of reticular thalamic nucleus (RTN) in WT and TREK1^−/−^ mice. ***B***, Bar graph compares the sIPSCs median amplitude between WT and TREK1^−/−^ RTN neurons. Cumulative probability plots of sIPSCs interevent interval recorded from RTN neurons of WT and TREK1^−/−^ mice. Bar graph compares the sIPSCs median interevent interval between WT and TREK1^−/−^ mice. ***C***, Cumulative probability plots of mIPSCs amplitude recorded in RTN. Bar graph compares the mIPSCs median amplitude between WT and TREK1^−/−^. ***D***, Cumulative probability plots of mIPSCs interevent interval recorded from RTN neurons in WT and TREK1^−/−^ mice. Bar graph compares the mIPSCs median interevent interval between WT and TREK1^−/−^ neurons. ***E–H***, Scatterplots display rise time, decay time, half-width, and charge transfer for individual cell data points between WT and TREK1^−/−^ RTN neurons, with the group mean indicated on the left *y*-axis. The difference between group medians (due to the non-normal dataset) is shown with a dashed line on the right *y*-axis. Error bars represent the SEM. ***I***, Simplified scheme of the stimulation electrode (SE) and recording electrode (RE) for recording of evoked IPSCs (eIPSCs) in VB TC neurons. As shown in (***I***) the stimulation electrode (bipolar tungsten) was positioned at the boundary between RTN and VB. ***J***, Example eIPSC traces recorded in WT and TREK1^−/−^ VB TC neurons. Recordings were performed in presence of DNQX and AP5. To confirm that the recorded response was a GABAergic response, bicuculline (20 µM) was administered at the end of recordings to the bath solution (right panel, TREK1^−/−^ black trace). ***K***, Bar graph comparing the paired-pulse ratios (PPRs) between WT and TREK1^−/−^ VB TC neurons. ***L***, Representative eIPSC traces are in presence of DNQX and AP5 (left panel, black trace, control condition) and after bath application of 10 µM spadin (right panel, blue trace) during VB TC neurons recording. To confirm that the recorded response was a GABA_A_-mediated current, bicuculline (20 µM) was administered at the end of recordings to the bath solution (right panel, green trace). Note how the decay time between conditions has changed. ***M***, Bar graph comparing the PPRs between control condition and after bath application of spadin 10 µM on VB TC neurons.

In addition, analysis of intra RTN synaptic activity did not show any difference in amplitude or frequency of IPSCs between TREK1 and WT RTN neurons (Fig. 5*A–D*). Comparing the mIPSCs kinetic characteristics in RTN neurons between TREK1^−/−^ and WT mice revealed a significant differences in mIPSCs rise time (Fig. 5*E*; 2.09 ± 0.1 ms, *n* = 8 cells, WT, vs 2.88 ± 0.16 ms, *n* = 9 cells, TREK1^−/−^, Mann–Whitney *U* test = 7, median difference = 0.93, *p* = 0.004), decay time (Fig. 5*F*; 4.63 ± 0.25 ms, *n* = 8 cells, WT, vs 6.34 ± 0.21 ms, *n* = 9 cells, TREK1^−/−^, Mann–Whitney *U* test = 0, median difference = 1.25, *p* < 0.0001), half-width (Fig. 5*G*; 3.28 ± 0.24 ms, *n* = 8 cells, vs 6.25 ± 0.48 ms, *n* = 9 cells TREK1^−/−^, Mann–Whitney *U* test = 0, median difference = 2.35, *p* < 0.0001), and charge transfer (Fig. 5*H*; 276.6 ± 23.3, *n* = 8 cells, WT, vs 413.16 ± 27, *n* = 9 cells, TREK1^−/−^, Mann–Whitney *U* test = 7, median difference = 124.6, *p* = 0.0037). These findings indicate that knock-out of TREK1 channels differentially affects inhibitory synaptic responses within RTN and its afferents to VB TC neurons.

To indicate whether lower IPSCs amplitude in TREK1^−/−^ VB TC neurons is due to alterations in release probability in presynaptic (RTN) neurons, IPSCs were evoked in VB neurons by local stimulation of RTN cells (Fig. 5*I–K*). Paired-pulse ratios (PPRs; second stimulus-evoked amplitude/first stimulus-evoked amplitude) were used to further study presynaptic alterations and short-term synaptic plasticity between WT and TREK1^−/−^ mice. TREK1^−/−^ VB TC neurons showed significantly higher PPRs after stimulation of RTN when compared with WT TC VB cells (0.81 ± 0.04, *n* = 12 cells, WT vs 0.95 ± 0.05, *n* = 10 cells, *t* = 2.1, df *=* 20, *p* = 0.049; Fig. 5*K*).

We further investigated these findings by acutely blocking TREK1 channels after bath application of 10 µM spadin. While PPR analysis showed no significant differences following spadin application (Fig. 5*L*,*M*), a clear slowing of the current decay was visible in the presence of spadin [15.36 ± 2.01 ms, *n* = 19 cells, control condition (DNQX + AP5) vs 27.57 ± 5.2 ms, *n* = 15 cells, spadin 10 µM, *t* = 2.4, df = 32, *p* = 0.023]. These findings indicate that spadin does not mimic the presynaptic effect of TREK1 knock-out on GABA stimulated synaptic responses. Further experiments will be needed to study spadin-mediated changes in postsynaptic GABA_A_ receptor kinetics.

Since the PPR is inversely correlated with activity-driven presynaptic GABA release, our results indicate that TREK1 channel deficiency decreased the probability of evoked IPSPs in RTN-VB synapses, without affecting spontaneous GABA presynaptic release. Furthermore, postsynaptic GABA_A_-mediated currents showed slower kinetics, which in turn would be altering signal processing of excitation/inhibition synaptic events at VB TC neurons.

### TREK1-deficient mice exhibit numerous sharp spindle oscillations during sleep

**Figure 6.**
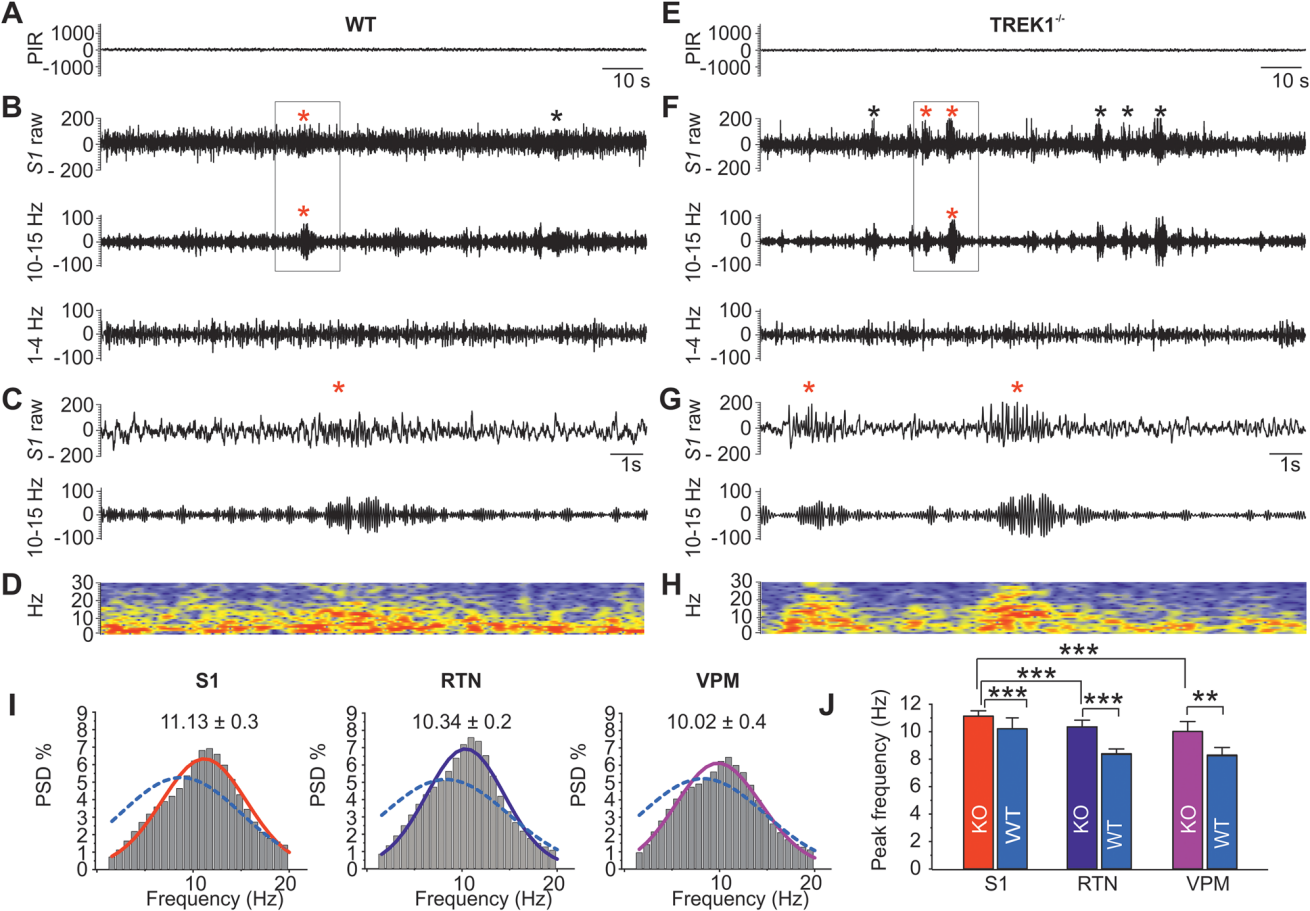
Appearance of numerous sharp spindle-like oscillations on local field potential (LFP) recordings of TREK1^−/−^ mice during episodes of sleep. ***A***, ***E***, The signal from a passive infrared movement detector (PIR) which in parallel with the local field potential (LFP) signal was used for detection of slow-wave sleep. Both slow-wave sleep and sleep spindles were visually defined after filtering the raw LFP signal with two separate filters for spindle (10–15 Hz) and delta (1–4 Hz) frequency oscillations. ***B***, Exemplary raw and filtered LFP traces from the somatosensory cortex (S1) of control mice containing typical sleep spindles (asterisks) which occur during non-rapid-eye-movement sleep (NREM). ***C***, Enlarged traces depicted in ***B*** (box containing red asterisk) with a corresponding time frequency graph analysis (***D***) for the raw LFP (bottom panel). ***F***, Exemplary filtered and raw LFP traces from the S1 of TREK1^−/−^ mice which show a cluster of sharp spindle-like oscillations (asterisks) appearing during episodes of slow-wave sleep. ***G***, Enlarged traces depicted in ***F*** (box) with a corresponding time frequency analysis graph (***H***) for the raw LFP (bottom panel). ***I***, Spindle peak frequency distributions represented for S1, RTN, and VPM using power spectral density analysis on 265 sleep spindles (*n* = 5, TREK1^−/−^ mice). Mean of peak frequencies were obtained after averaging the Gaussian distribution of spindles for all five animals and are denoted in the top right corner of each graph. For comparison, the spindle peak frequency distributions for S1, RTN, and VPM of WT mice (100 sleep spindles, *n* = 5) are over imposed on the frequency plots in ***I*** as dashed lines. ***J***, Bar graphs comparing the frequency of the peak of spindles in S1, RTN and VPM between and within genotypes.

Together with HCN channels, TREK and TASK channels contribute to RMP of TC neurons and govern switching between tonic and burst firing modes and therefore are important in generation of thalamocortical oscillations during sleep and wakefulness (Meuth et al., 2006; Bista et al., 2012). In addition, burst firing in RTN neurons results in the generation of sleep spindles underlined by synaptic inhibition of TC relay cells (Steriade, 2005). Burst firing is highly dependent on membrane potential and happens at potentials negative to −55 mV. Therefore, leak potassium channels that contribute to the hyperpolarized RMP of RTN neurons can potentially influence the generation of sleep spindles. Interestingly, the cortical LFP in TREK1^−/−^ mice showed a marked difference in the number and morphology of the sleep spindles. Typical examples of sharp sleep spindles are illustrated in Figure 6*E*–*H*. The spindles in TREK1^−/−^ mice occurred mostly in clusters and were different in appearance (sharper instead of being sinusoidal) from conventional sleep spindles found in WT animals (Fig. 6*A–D*). To characterize these spindles in TREK1^−/−^ mice, both slow-wave sleep and sleep spindles were visually defined after filtering the raw LFP signal with two separate bandpass filters for spindle (10–15 Hz) and delta (1–4 Hz) frequency oscillations. Spindles were selected based on similar characteristics as typical sleep spindles including 10–12 Hz waxing and waning waveforms with amplitude at least two times larger than the background LFP amplitude. The number of spindles was counted for total duration of 10 min of slow-wave sleep (selected from the first 2 h of light period) for each animal and was averaged for each group. TREK1^−/−^ showed a significantly higher number of spindles in cortical LFP compared with WT mice (42.1 ± 2.9, *n* = 5, TREK1^−/−^ mice vs 18.8 ± 3.1, *n* = 5 WT mice, *t* = 5.54, df = 8, *p* = 0.001). However, the mean duration of sleep spindles was similar between the two groups (S1: 1.71 ± 0.1 s, *n* = 100 spindles/5 WT mice vs 1.9 ± 012 s, *n* = 256 spindles/5 TREK1^−/−^ mice). Peak frequency distribution of cortical (S1) and thalamic (RTN and VPM) spindles were calculated by performing normalized power spectral density (PSD%) analysis on 265 sleep spindles selected from 5 TREK1^−/−^ mice and 100 sleep spindles from WT mice (*n* = 5 mice per genotype; Fig. 6*I*). The peak frequencies were obtained after fitting the data with Gaussian distribution and were 11.13 ± 0.3 TREK1^−/−^ versus 9.3 ± 0.3 WT for S1, 10.34 ± 0.2 TREK1^−/−^ versus 8.4 ± 0.2 WT for RTN, and 10.02 ± 0.4 TREK1^−/−^ versus 8.3 ± 0.22 for VPM (Fig. 6*I*,*J*). Figure 6*J* illustrates the differences in the peak frequency of sleep spindles between and within genotypes for the three regions in bar graphs. Compared with the peak frequency of sleep spindles in WT mice, the sharp sleep spindles in TREK1^−/−^ mice showed higher frequencies for all the three regions (repeated-measures ANOVA followed by Student's *t* tests as post hoc revealed a significant within subject effect for regions and a significant main effect for genotypes, *p* < 0.05). In TREK1^−/−^ mice significant differences were found in peak frequency of spindles when S1 was compared with both RTN (paired-samples Student's *t* test, *t* = 9.5, df = 4, *p* < 0.001) and VPM (paired-samples Student's *t* test, *t* = 9.9, df = 4, *p* < 0.001). The peak frequency of spindles between RTN and VPM was not significantly different. There were no significant differences between the peak frequency of spindles between S1, RTN, and VPM in WT mice.

These findings point to a contribution of TREK1 in the generation of sleep spindle, as well as in shaping the morphology of sleep spindles. This is probably due a dynamic interaction between its influence on membrane potential and synaptic properties of RTN neurons with their fine-tuning ability by ACh and other neurotransmitters during sleep–wake transitions.

### Changes in thalamocortical oscillations and sleep–wake behavior in TREK1^−/−^ mice

**Figure 7.**
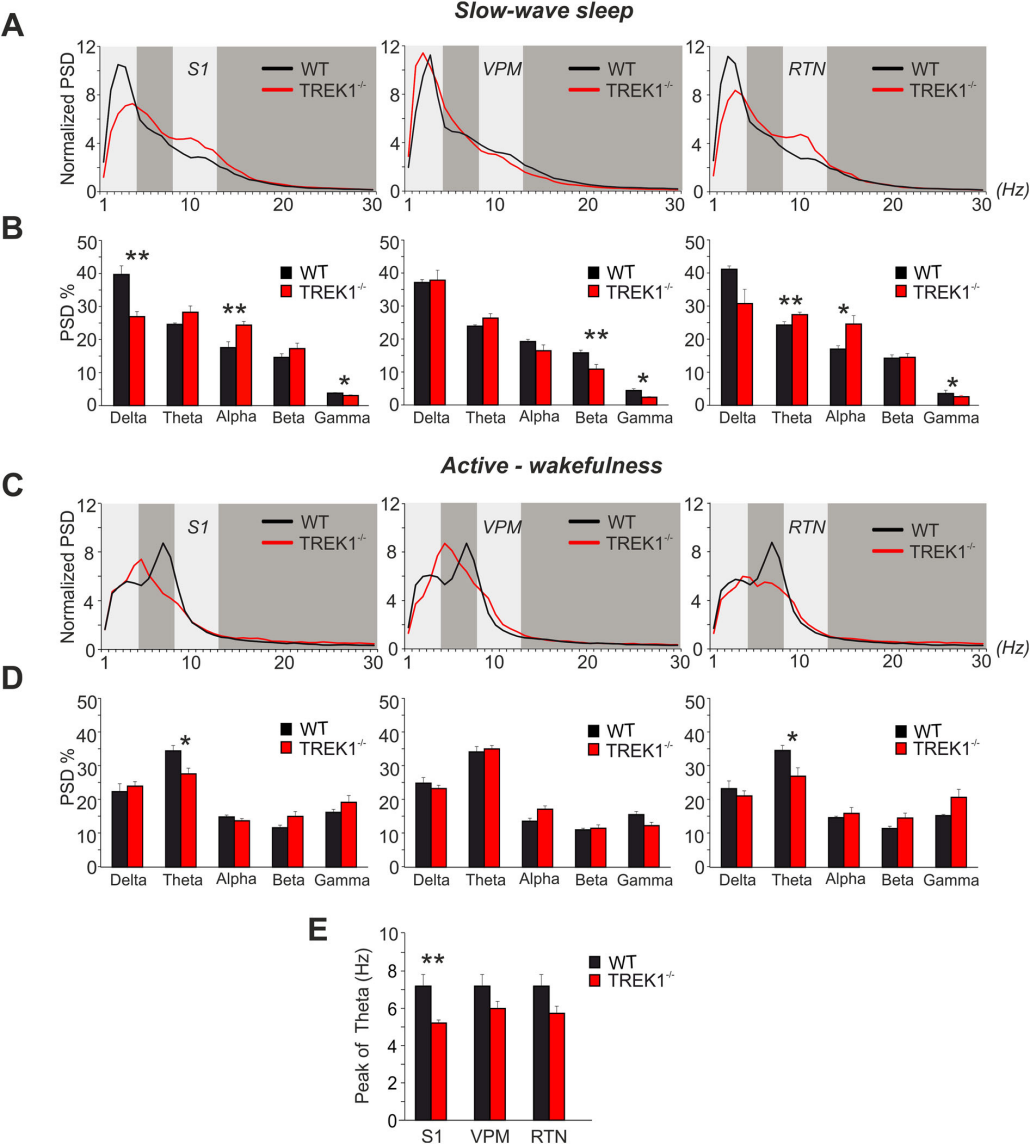
Effects of TREK1 on thalamocortical oscillatory activity. Analysis of power spectral density (PSD) was performed for frequencies between 1 and 100 Hz on somatosensory cortex (S1), ventral posterior medial (VPM) nucleus of the thalamus, and reticular thalamic nucleus (RTN). Only the first 2 h of light period were used for PSD analysis. ***A***, Spectrograms compare the mean power of spectral density (normalized) for delta (1–4), theta (4.5–8), alpha (8.5–12), and beta (13–30) bands in the S1, VPM, and RTN during slow-wave sleep between TREK1^−/−^ (in red) and WT (in black) mice. For clarity, only frequencies between 1 and 30 Hz are presented in graphs. ***B***, Bar graphs compare the total percentage of PSD (PSD%) during slow-wave sleep between WT and TREK1^−/−^ mice. ***C***, Spectrograms comparing the mean power of spectral density (normalized) for frequencies between 1 and 30 Hz during episodes of active-wakefulness between TREK1^−/−^ (in red) and WT (WT) mice (in black). ***D***, Comparison of the total percentage of PSD% during active-wakefulness between WT and TREK1^−/−^ mice. ***E***, Comparing the frequency of the peak of theta during active-wakefulness between WT and TREK1^−/−^ in S1, VPM, and RTN.

Given the importance of TREK1 channels in regulation of passive and active membrane properties of thalamic neurons and synaptic communication between RTN and VB, we next assessed the effect of TREK1 channel deletion on thalamocortical network oscillations (Fig. 7). PSD analysis was performed on S1, VPM, and RTN for frequencies between 1 and 100 Hz. Point to point comparison of the normalized PSDs during slow-wave sleep between the two genotypes (Fig. 7*A*) revealed a significant reduction in delta frequency oscillations (1–4 Hz) in TREK1^−/−^ LFP for S1 (*F*_(37,296)_ = 8.34, *p* = 0.000) and RTN (*F*_(37,296)_ = 2.53, *p* = 0.000) and an increase in theta band oscillations. Less pronounced differences in sleep LFP of TREK1^−/−^ and WT mice were recorded from VPM (Fig. 7*A*, middle panel). Comparison of the total percentage of PSD (PSD%) during slow-wave sleep between WT and TREK1^−/−^ in cortex (S1) and in the thalamus (RTN and VPM) demonstrated a significantly lower power for delta in S1 (26.93 ± 1.54 TREK1^−/−^ vs 39.7 ± 2.62 WT); for gamma in S1 (3.04 ± 0.2 TREK1^−/−^ vs 3.8 ± 1.16 WT), in VPM (2.3 ± 0.2 TREK1^−/−^ vs 4.5 ± 0.5 WT), and in RTN (2.6 ± 0.35 TREK1^−/−^ vs 3.6 ± 0.04 WT); and for beta in VPM (10.856 ± 1.4 TREK1^−/−^ vs 15.8 ± 0.8 WT) in TREK1^−/−^ mice compared with WT (mixed-repeated-measures ANOVA's followed by Student's *t* tests, *p*s < 0.05, *n* = 5/5; Fig. 7*B*). In addition, TREK1^−/−^ showed a significantly higher PSD for theta in RTN (27.27 ± 0.74 TREK1^−/−^ vs 24.27 ± 0.07 WT) and for alpha in both RTN (24.6 ± 2.55 TREK1^−/−^ vs 16.9 ± 1.12 WT) and S1 (24.4 ± 1.12 TREK1^−/−^ vs 17.6 ± 1.8 WT).

Comparing the normalized PSD during active-wakefulness between TREK1^−/−^ and WT mice revealed a significant decrease in the power of theta in S1 (*F*_(37,296)_ = 2.7, *p* = 0.000) and RTN (*F*_(37,296)_ = 3.2, *p* = 0.000; Fig. 7*C*). This was further confirmed when the total theta PSD% was compared between the two groups in S1 (27.7 ± 1.7 TREK1^−/−^ vs 34.51 ± 1.6 WT) and RTN (27.09 ± 2.5 TREK1^−/−^ vs 34.75 ± 1.5 WT; Fig. 7*D*). No significant changes in the power of theta were found in VPM of TREK1^−/−^ compared with WT mice. Interestingly, the peak of the theta band was shifted to lower frequency values in all three regions. Comparing the peak of theta band between TREK1^−/−^ and WT mice revealed a significant leftward shift for S1 (5.21 ± 0.2 Hz TREK1^−/−^ vs 7.2 ± 0.62 Hz WT; Fig. 7*E*).

**Figure 8.**
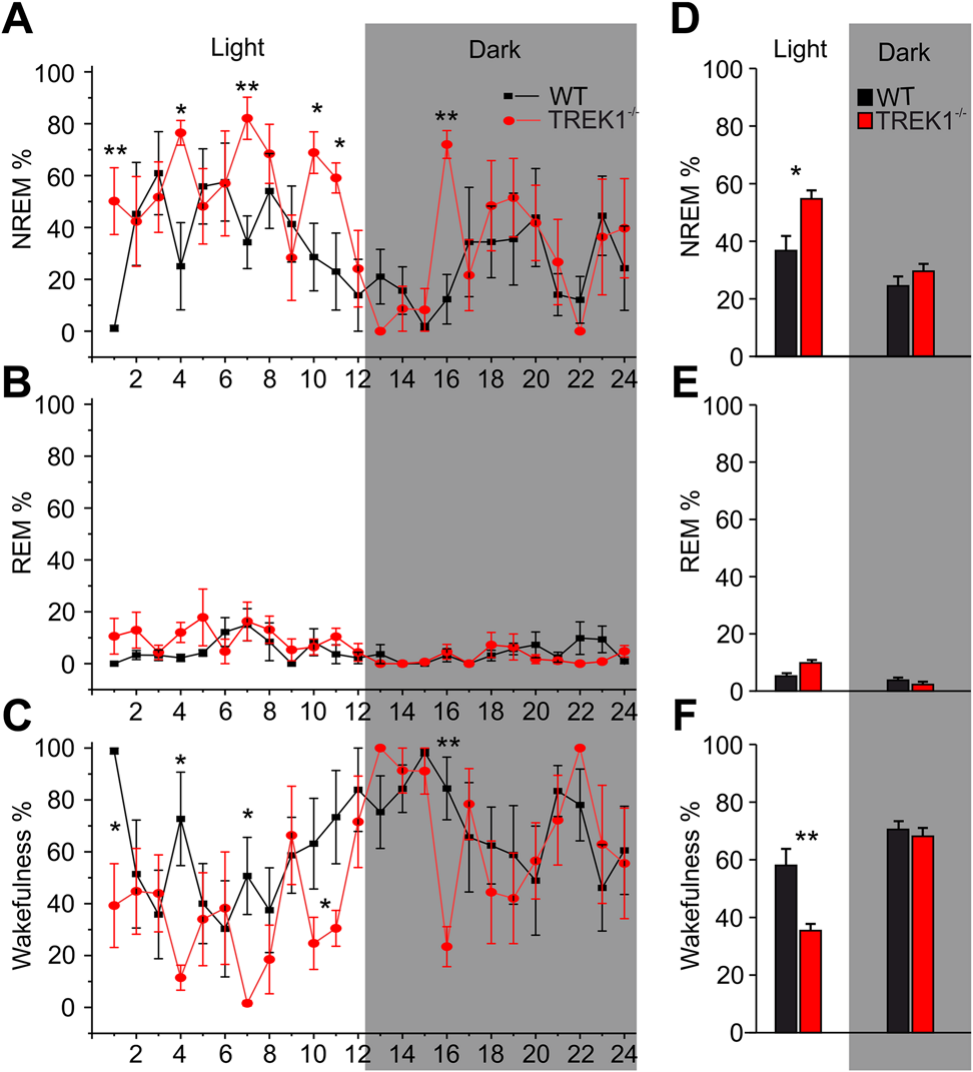
Absence of TREK1 potassium channels alter sleep and wake transitions in TREK1^−/−^ mice. Different states of vigilance including non-rapid-eye-movement sleep (NREM), rapid-eye-movement sleep (REM), and wakefulness were scored manually by a trained individual. Sleep–wake scoring was performed on the first 10 min of every hour from 24 h LFP recording (total of 240 min LFP data per animal). ***A–C***, Comparison of the hourly percentage of NREM (***A***), REM (***B***), and wakefulness (***C***) between WT (*n* = 5 mice) and TREK1^−/−^ (*n* = 5) mice revealed an increase in NREM% (***A***) and a reduction Wakefulness% (***C***) in TREK1^−/−^ compared with WT mice. ***D–F***, Bar graphs comparing the percentage of total time spent in each of the three states during the 240 min (120 min light cycle/120 min dark cycle) of recording period between WT and TREK1^−/−^ mice. Repeated-measures ANOVA revealed a time-of-day-dependent alteration in the amount of sleep and wakefulness in TREK1^−/−^ mice, with a significant increase in total percentage of NREM sleep (***D***) and relative reduction in percentage of wakefulness during light cycle (***F***). No significant changes in the amount of REM sleep were observed in TREK1^−/−^ mice.

Both dorsal and ventral thalami play important roles in sleep. To understand the possible role of TREK1 in sleep behavior, we compared the sleep and wake cycle of the two groups. Different states of vigilance including NREM, REM, and wakefulness were scored manually by a trained individual. Sleep–wake scoring was performed on the first 10 min of every hour from 24 h LFP recordings (total of 240 min LFP data per animal). As shown in Figure 8*A–C*, comparison of the hourly percentage of NREM, REM, and wakefulness between WT and TREK1^−/−^ mice revealed an increase in the percentage of NREM sleep (NREM%, repeated-measures ANOVAs, significant time of day effect; *F*_(23,184)_ = 3.1, *n* = 5 mice, *p* = 0.000, and significant main effect for genotypes; *F*_(1,8)_ = 8.61, *n* = 5 mice, *p* = 0.019) and a reduction in percentage of wakefulness (Wakefulness%, repeated-measures ANOVAs, significant time of day effect; *F*_(23,184)_ = 3.4, *p* = 0.000, and significant main effect for genotypes; *F*_(1,8)_ = 12.75, *p* = 0.019, *n* = 5/5 mice) in TREK1^−/−^ compared with WT mice. Next, the percentage of total time spent in each of the three states of vigilance during the light (120 min duration) and dark (120 min duration) hours were compared between TREK1^−/−^ and WT mice (time of the day as within-subjects factor and genotypes as between-subjects factor). Repeated-measures ANOVA revealed a time-of-day-dependent alteration in the amount of sleep and wakefulness in TREK1^−/−^ mice (significant interaction between state of vigilance, time of day, and genotypes, *F*_(2,16)_ = 5.7, *p* = 0.014, *n* = 5/5 mice), with a significant increase in total percentage of NREM sleep (*t* = 3.06, df = 8, *p* = 0.016, *n* = 5/5) and relative reduction in percentage of wakefulness (*t* = 3.6, df = 8, *p* = 0.006) during light cycle (Fig. 8*D*,*F*). No significant difference between TREK1^−/−^ and WT mice in the amount of REM sleep was observed (Fig. 8*E*).

### Mathematical modeling

**Figure 9.**
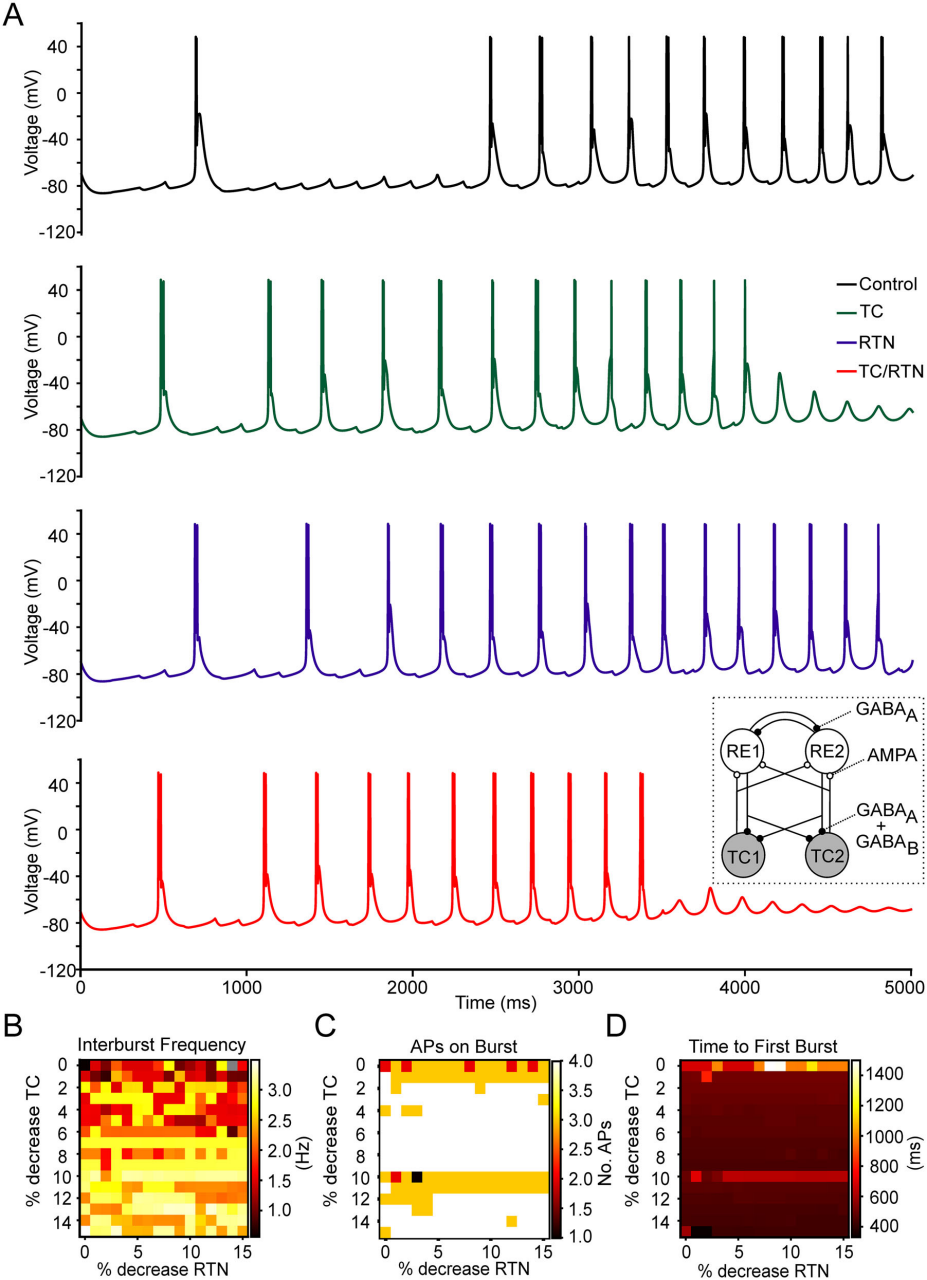
Simulations showing the effects of reducing potassium leak conductance on TC spindle oscillations in a four-neuron circuit of TC and RTN neurons. ***A***, Example voltage traces of a TC cell under control conditions (black trace) and when the maximum potassium leak conductance is decreased by 5% in TC (green trace), RTN cells (blue trace), and both TC and RTN cells (red trace). The inset illustrates the mathematical model of the intrathalamic network using a four-cell model system (Chaudhary et al., 2022). Heat maps of the interburst frequency (***B***), number of action potentials in a burst (***C***), and the time to the first spike (***D***) in a TC cell as a function of a decrease in the percent maximum leak conductance in TC cells (vertical direction) and in RTN cells (horizontal direction).

To assess the impact of reducing the background leak conductance in TC and RTN neurons on spontaneous bursting, a computational model was used (Fig. 9*A*, inset). With the K^+^ leak conductances in each cell module set to the build in default value, rhythmic bursting in the delta range was found (Fig. 9*A*, black trace). In order to assess a large parameter space, the default value was systematically reduced in 1% decrements up to −15% in TC (Fig. 9*A*, green trace) or RTN (Fig. 9*A*, blue trace) cells alone or in both cell types concordantly (Fig. 9*A*, red trace). The resulting heat maps indicate subtle effects of K^+^ leak reduction on the interburst frequency (Fig. 9*B*), the number of APs per burst (Fig. 9*C*), and the time to the first AP (Fig. 9*D*). Overall, the frequency of delta oscillations (more yellowish in the lower half of Fig. 9*B*) and the number of APs per burst seem to slightly increase (domination of white in Fig. 9*C*) with less K^+^ leak conductance. The start of the oscillation seems to be less influenced (Fig. 9*D*). These data indicate that the reduction of leak conductances in thalamic neurons changes rhythmic oscillations.

### TREK1 deletion does not alter mRNA expression of other ion channels

To investigate whether TREK1 knock-out during early developmental stages impacts the transcriptional regulation of other ion channels, quantitative PCR (qPCR) analyses were conducted across multiple brain regions in TREK1^−/−^ mice and WT controls (data not shown). The mRNA expression levels of voltage-gated sodium channels (Scn1a, Scn3a, and Scn8a), TRPM4 (transient receptor potential cation channel subfamily M member 4), TREK2, and GABA_A_ receptor subunits (Gabra1, Gabrb2, Gabrg2, Gabra4, and Gabrd) were quantified in the somatosensory cortex (S1), RTN, A1, hippocampus (HC), and VB. Comparative analysis revealed no statistically significant differences in mRNA expression between WT and TREK1^−/−^ mice, suggesting that the absence of TREK1 does not disrupt the transcriptional homeostasis of these channels.

### Increased excitability in TREK1^−/−^ pyramidal neurons in the somatosensory cortex

**Figure 10.**
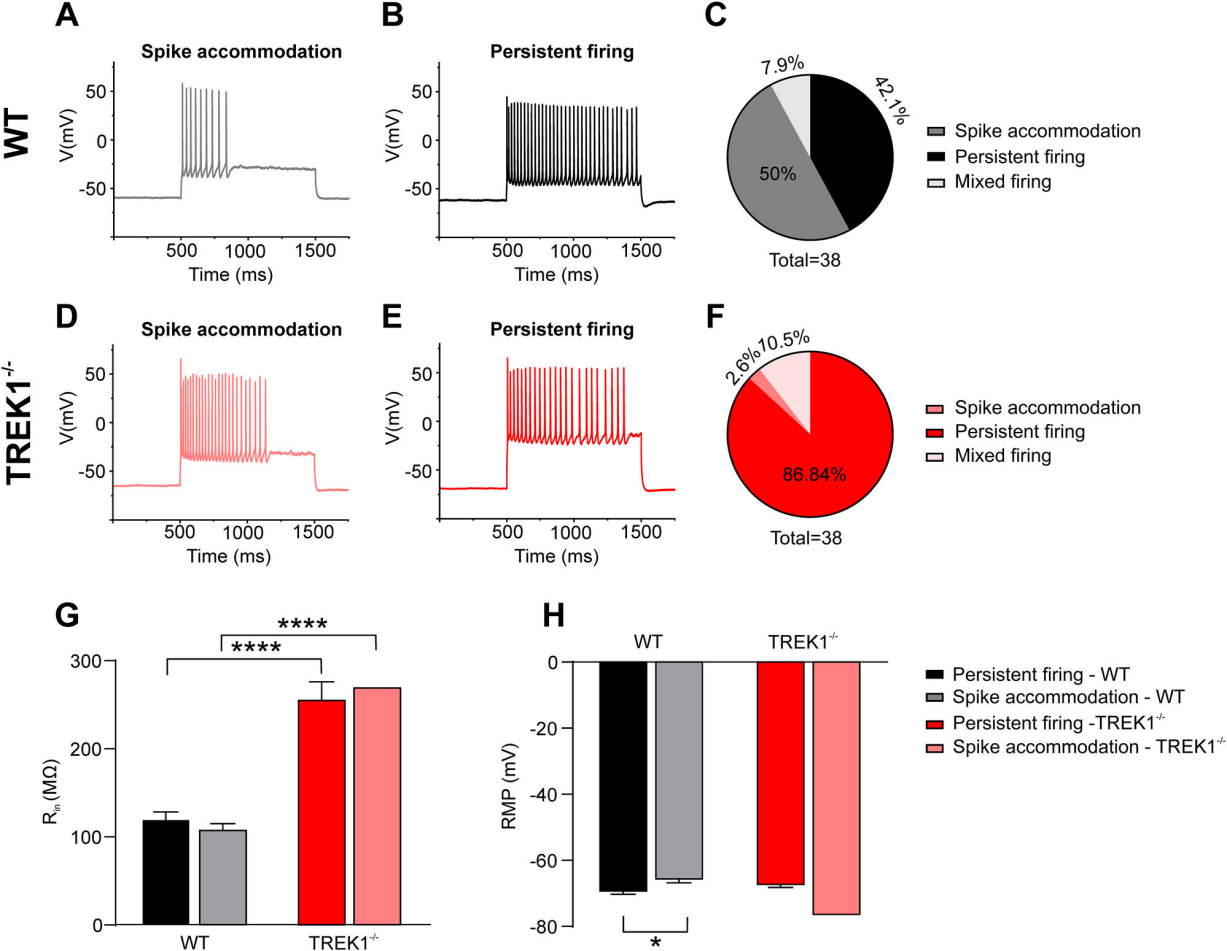
Increased excitability in TREK1^−/−^ pyramidal neurons in the somatosensory cortex. ***A***, ***B***, Representative traces of action potential (AP) firing modes in pyramidal neurons of layers 2–4 in the somatosensory cortex (S1) of WT mice, showing two firing types: spike accommodation (***A***) and persistent firing (***B***). These responses were elicited by 1 s depolarizing current pulses (+420 pA) after adjusting the resting membrane potential (RMP) to −55 mV. ***C***, Pie chart showing the distribution of AP firing modes in S1 pyramidal neurons of WT mice, with color coding for spike accommodation, persistent firing, and mixed firing modes. ***D***, ***E***, Representative traces of spike accommodation (***D***) and persistent firing (***E***) AP modes in pyramidal neurons of layers 2–4 in S1 of TREK1^−/−^ mice, elicited by 1 s depolarizing current pulses (+420 pA) with RMP adjusted to −55 mV. ***F***, Pie chart showing the distribution of AP firing modes in S1 pyramidal neurons of TREK1^−/−^ mice, with color coding for spike accommodation, persistent firing, and mixed firing modes. ***G***, Graph comparing input resistance (*R*_in_) of S1 neurons between WT and TREK1^−/−^ mice across the two AP firing modes, spike accommodation, and persistent firing. ***H***, Bar graph comparing resting membrane potential (RMP) between WT and TREK1^−/−^ mice for the two AP firing modes.

To investigate the effects of TREK1 deletion on neuronal excitability in cortical pyramidal neurons, AP firing patterns were analyzed in visually identified pyramidal cells from layers 2–4 of the somatosensory cortex (Fig. 10). In TREK1^−/−^ neurons, 86.8% exhibited persistent firing, a mode characterized by continuous action potential firing, indicative of increased excitability. In contrast, only 42.1% of WT neurons displayed persistent firing. Furthermore, 50% of WT neurons showed spike frequency accommodation, a firing mode restricting long-lasting repetitive AP generation, whereas only 2.6% of TREK1^−/−^ neurons exhibited spike frequency accommodation. Additionally, 10.5% of TREK1^−/−^ neurons displayed mixed firing patterns (Fig. 10*A–F*).

Analysis of *R*_in_ further revealed higher values in both persistent firing neurons (121.4 ± 10.1 MΩ, *n* = 16 cells WT vs 257.2 ± 21.2 MΩ, *n* = 33 cells TREK1^−/−^, *t* = 4.3, df = 47, *p* < 0.0001) and spike frequency accommodating (109.3 ± 8.6 MΩ, *n* = 19 cells WT vs 271.1 ± 0.0 MΩ, *n* = 1 cells TREK1^−/−^, *t* = 4.2, df = 18, *p* < 0.0001) neurons in TREK1^−/−^ mice compared with their respective counterparts in WT mice (Fig. 10*G*). These results suggest that TREK1 deletion increases neuronal excitability, as evidenced by both the altered firing patterns and the elevated input resistance of these neurons.

### TREK1-deficient mice exhibit a robust neuronal enhancement in auditory thalamocortical system

Since clusters of RTN neurons that receive auditory inputs interact with TC neurons of the vMGN (Shosaku and Sumitomo, 1983) and altered TREK1 channel expression in the auditory system contributes to activity-dependent plasticity after bilateral cochlear ablation (Holt et al., 2006), we evaluated the possibility that the absence of TREK1 channels in RTN and vMGN might influence sensory information processing.

**Figure 11.**
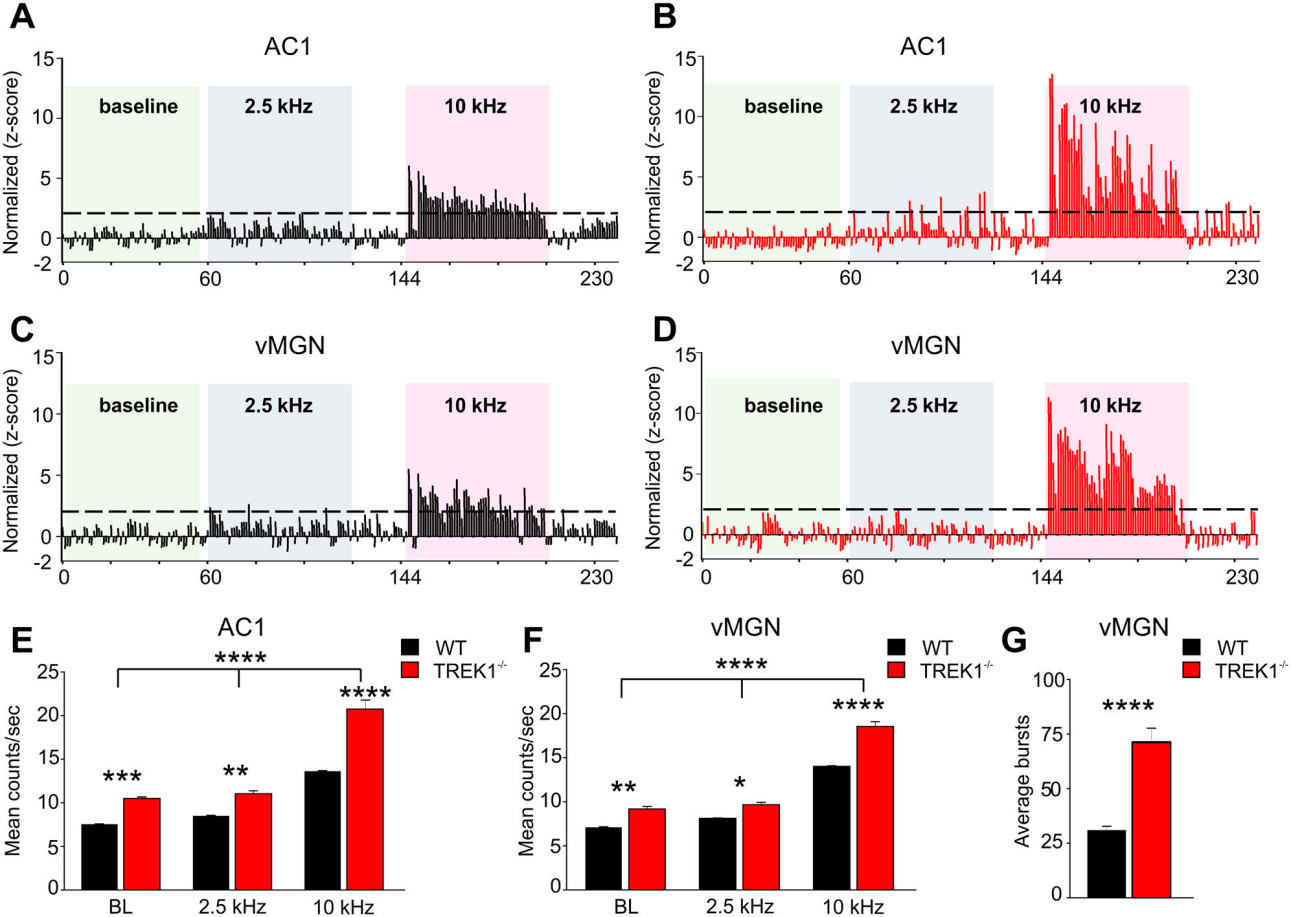
TREK1 deletion affects the tone-induced neuronal activity in vivo. Histograms illustrating the frequency-dependent response to a randomly presented series of low (2.5 kHz) and high (10 kHz) frequencies in WT and TREK1^−/−^ animals recorded from layer IV of the auditory cortex (AC1) and ventral part of the medial geniculate nucleus (vMGN). ***A***, ***B***, Typical rate histograms of the overall neuronal responses of AC1 in WT (***A***) and TREK1^−/−^ (***B***) mice during the recording time period. The overall AC1 activity is significantly increased in TREK1^−/−^ compared with WT (***A***) mice. ***C***, ***D***, The overall vMGN activity is significantly increased in TREK1^−/−^ (***D***) compared with WT (***C***). ***E***, Quantification of the AC1 neuronal activation in response to the presentation of auditory stimuli of 2.5 or 10 kHz in TREK1^−/−^ and WT. ***F***, Quantification of the vMGN neuronal activation in response to the presentation of auditory stimuli of 2.5 or 10 kHz in WT and TREK1^−/−^. ***G***, Deletion of TREK1 altered the bursting activity in vMGN. The percentage of burst spikes that occurred in the vMGN of TREK1^−/−^ was significantly increased compared with the WT mice.

To determine how TREK1 deletion affects neuronal excitability, electrodes were placed in both the vMGN and layer 4 of the AC1 (Cerina et al., 2017). Auditory stimuli at 2.5 and 10 kHz were presented at different durations in a pseudorandomized order and neuronal responses in vMGN and AC1 were recorded as single unit activity. Based on their *z*-score values, we analyzed neurons which represented significant activation upon presentation of auditory stimuli in comparison with their baseline activity; neurons with a response that was 1.96-fold higher than the baseline: *z*-score ≥1.96 to baseline, *p* ≤ 0.05 indicated by dashed lines in Figure 11*A–D*. TREK1^−/−^ mice showed an increase in *z*-score in response to 10 kHz compared with WT mice, corroborating the frequency-specific characteristics of recording sites in the AC1 and vMGN (Fig. 11*B*,*D*, red traces). These results were confirmed by stimulus-related firing rates obtained by averaging auditory-dependent responses. Compared with WT mice, TREK1^−/−^ revealed a significantly higher response magnitude at 10 kHz both in AC1 (two-way ANOVA, *F*_(2,198)_ = 17.59, *p* = 0.0001; Fig. 11*E*) and vMGN (*F*_(2,198)_ = 8.775, *p* = 0.0001; Fig. 11*F*). Additionally, the stimulus-related firing rates revealed that neuronal responses to both baselines and at 2.5 kHz were significantly higher in TREK1^−/−^ compared with the WT (Fig. 11*E*,*F*).

Next, the prototypical thalamic activity modes of burst firing were assessed by analyzing overall single unit activity as described before (Narayanan et al., 2018). The firing pattern of vMGN neurons, namely, the average of burst firing, was strongly altered after TREK1 deletion (*t* = 6.8, df = 20, *n* = 21 neurons, *p* = 0.0001; Fig. 11*G*).

Since the deletion of TREK1 heavily affects neuronal excitability, we investigated the firing latencies and found no significant difference between TREK1^−/−^ and WT mice (data not shown).

These findings indicate that TREK1 channels limit basal and sensory-induced neuronal activity in the auditory pathway.

## Discussion

### The contribution of TREK1 channels to cellular activity in the thalamus

In RTN neurons, TREK1 channels contribute to setting the range of negative RMP, presynaptic transmitter release, and the properties and generation of tonic AP. The latter is essential as tonic firing was nearly abolished in TREK1^−/−^ mice. That these changes indeed depend on functional TREK1 channels was proved using spadin, which largely replicated the results of channel knock-out, showing a key mechanism in thalamocortical networks that cannot be compensated by other ion channels. RTN cells express a unique combination of ion channels that regulate their spike output (Fernandez and Lüthi, 2020). A short depolarizing pulse from the RMP activates low-threshold Ca_V_3 channels, leading to the generation of an initial burst discharge. Consequently, Ca^2+^- dependent K^+^ channels of the SK2-type and nonselective cation currents (*I*_CAN_) generated by transient receptor potential cation channel subfamily M member 4 (TRPM4) channels are activated by the Ca^2+^ ions entering through Ca_V_3 channels (Cueni et al., 2008; O’Malley et al., 2020). The activation of the depolarizing channels triggers long-lasting plateau potentials and persistent tonic firing with SK channels controlling the duration of firing. Since the RMP in RTN neurons in TREK1^−/−^ mice is more depolarized, the driving force for Ca^2+^ influx through Ca_V_3 channels may be insufficient for full TRPM4 activation. Consequently, the long-lasting plateau potential mediated by TRPM4 channels is smaller and a reduced number of APs is generated. We want to emphasize that the firing pattern of RTN neurons is more heterogeneous as delineated by this scenario (Clemente-Perez et al., 2017). Especially, the magnitude of Ca_V_3-mediated currents, likely a critical factor in determining the incidence of persistent firing in each neuron, varies. In addition, K_V_3.1, K_V_3.3 (Espinosa et al., 2008), Ca_V_2.3 (Weiergräber et al., 2008), and Na_V_1.1 (Kalume et al., 2015) channels have important roles in shaping repetitive firing. Indeed, APs of RTN have a wide operational range of voltage thresholds due to the availability of intrinsic Na_V_ channels as a result of the preceding membrane potential (Muñoz and Fuentealba, 2012). However, AP threshold was not different between WT and TREK^−/−^ mice in the present study. Since TREK1 channels support sustained AP firing (Corbin-Leftwich et al., 2018), even in the absence of K_V_ channels (MacKenzie et al., 2015), new data showing changes in shape and kinetics of the action potentials of RTN and VB neurons suggest that the absence of these channels may disturb the complex interplay of ion channels.

While depolarization-induced firing patterns were less affected by TREK1 loss in VB TC neurons, AP duration was altered. Computational modeling shows that AP rise speed correlates with TREK1 conductance (MacKenzie et al., 2015). Indeed, the loss of TREK1 was correlated with a slower AP upstroke. The lack of further changes in VB TC neurons compared with RTN neurons may reflect nucleus-specific roles and/or compensatory mechanisms. Indeed, TREK1 knock-out or spadin application affects TC neuron firing in vMGN (present study) and dLGN (Bista et al., 2015).

Additionally, we found that Kir channel function is increased in VB TC neurons (data not shown), providing additional hyperpolarizing current. Notably, the compensatory capacity in VB TC neurons has been observed previously (Brickley et al., 2001; Labbaf et al., 2023). Nevertheless, quantitative expression profiling of selected voltage-dependent and ligand-gated ion channels, along with TREK2, showed no significant mRNA expression differences in five brain regions (data not shown), suggesting no extensive compensatory gene expression in TREK1^−/−^.

Specific K^+^ channels are vital for auditory processing (Kharkovets et al., 2000; Karschin et al., 2001). Our study highlights TREK1 channels’ role in modulating vMGN neuron activity in response to 10 kHz tones. TREK1 knock-out increased basal firing, tone-induced responses, bursting, and slightly altered frequency discrimination in the auditory thalamus and cortex. K^+^ channels exert important functional roles in the auditory system since auditory neurons adjust K_V_3 channel expression to fine-tune their firing (Li et al., 2001). In addition, K_V_7 activation by retigabine reverses frequency discrimination loss in neuroinflammation (Kapell et al., 2023). TREK1 activators may offer similar benefits (Schroeter et al., 2023).

### The contribution of TREK1 channels to synaptic activity in the thalamus

The RTN is the major source of inhibition in the thalamus, and RTN-mediated synaptic inhibition is essential for thalamic spindle activity (Fernandez and Lüthi, 2020). RTN neuron AP firing provides reliable, powerful synaptic inhibition to various TC cells. However, the reciprocal connections between the glutamatergic TC and GABAergic RTN neurons form open- rather than closed-loop connections (Pinault, 2011). TC cells express postsynaptic GABA_A_ receptors containing α1, β2, and γ2 subunits leading to large IPSPs following opening (Ulrich and Huguenard, 1997; Jia et al., 2009). Upon bursting and longer-lasting AP generation, GABA spills over the synaptic cleft, leading to the recruitment of nonsynaptic, α4-containing, and δ-containing GABA_A_ receptors, leading to larger and slower IPSPs (Herd et al., 2013). The current study's synaptic recordings provide insights into the contribution of TREK1 channels, as no changes in mRNA expression levels of various GABA_A_ receptor subunits were found in TREK1^−/−^ mice (data not shown). Recordings of mIPSCs in TC cells in the presence of TTX revealed no differences in amplitude and frequency between TREK1^−/−^ and WT mice, thereby indicating that the quantal neurotransmitter size was unchanged (Lim et al., 1999). However, we have observed slower kinetics for mIPSCs in TC cells from knock-out mice, suggesting that compensatory changes at the postsynaptic GABA_A_ receptor level were triggered by the absence of TREK1 channels.

In the absence of TTX, sIPSCs resulting from spontaneously occurring APs revealed significantly reduced amplitudes in TREK1^−/−^ mice, indicating that TREK1 channels control the release of GABA at RTN-VB presynaptic terminals induced by spontaneous AP firing.

Synaptic depression upon repeated use is a common form of short-term plasticity that can last from seconds to minutes (Saviane et al., 2002). Consequently, the PPR reaches values <1.0 in paired-pulse protocols. Although other mechanisms may contribute, this depression mainly reflects the depletion of a readily releasable pool of vesicles. The finding that the PPR is inversely related to the initial release probability (Dobrunz and Stevens, 1997) is in line with our results showing that TREK1^−/−^ mice have decreased spontaneous GABA release, which in turn reduces the probability of evoked IPSCs in RTN→VB synapses. Again, a reduction in the driving force for Ca^2+^ influx may play a role. It should be noted that acute spadin application had effects different from TREK1 channel knock-out, thereby pointing to alterations in mechanisms and components of synaptic transmission not investigated here.

### Contribution of TREK1 channels to spindling in the thalamus

Spindle oscillations are generated through a combination of intrinsic mechanisms, like Ca_V_3 channel-dependent bursting in RTN neurons and the synaptic interactions between RTN and TC neurons. Based on previous experimental and modeling studies, it has been suggested that ∼10 Hz rhythms are intrinsic to reticular thalamic networks (Iavarone et al., 2023). This aligns well with our in vivo analysis of spindle frequency bands. A novel thalamo-reticular circuit model recently indicated that previously unappreciated synaptic mechanisms, including release probability and short-term synaptic depression, play a role in shaping spindle-like oscillations (Iavarone et al., 2023). Especially the waning of the spindle-like oscillation depends on the progressive reduction in the probability of synaptic release due to short-term synaptic depression at RTN→TC cell synapses. Therefore, the generation of sharp spindle oscillations in TREK1^−/−^ mice may be based on alterations in both parameters, namely, release probability and short-term synaptic depression. In addition, the RMP of RTN and VB TC neurons influences the frequency of spindle-like oscillations in the thalamo-reticular circuit (Iavarone et al., 2023). In general, VB TC neuron depolarization increases duration and frequency, while RTN cell depolarization decreases them. However, specific combinations of both parameters result in increased frequencies when RTN cells depolarize and VB TC neurons reveal unchanged RMP. These findings may provide the computational ground for the increase in spindles and their change in morphology toward the occurrence of sharp spindle waves in TREK1^−/−^ mice.

Blocking GABAergic inhibition in thalamic slices with bicuculline slows 6–10 Hz spindle oscillations (Von Krosigk et al., 1993). Similarly, reduced IPSP amplitudes in RTN neurons of TREK1^−/−^ mice are linked to slower theta oscillations in the awake state. Since cortical activity heavily influences sleep oscillations (Krone et al., 2021), altered cortical excitability may drive sharp spindle generation. Analysis of AP firing in S1 pyramidal cells (layers 2–4) shows increased excitability in TREK1^−/−^ mice. As cortical activity controls fast spindles (Timofeev and Chauvette, 2013), increased cortical activity may explain the faster sharp spindle waves in TREK1^−/−^. These findings indicated that cortical TREK1 channels contribute to our in vivo findings. Future studies have to unravel the function of cortical TREK1 channels.

It could be considered to test the role of TREK1 channels on brain oscillations using pharmacological tools in vivo. However, in line with the 3R principles, in this study, we decided to use a mathematical modeling approach to reduce reliance on animal testing, which is becoming increasingly difficult in the European Union, particularly in Germany (Schröder et al., 2024). Indeed, our computer model shows that reducing K^+^ leak channels affects slow intrathalamic burst firing.
